# Alloimmunity to Class 2 Human Leucocyte Antigens May Reduce HIV-1 Acquisition – A Nested Case-Control Study in HIV-1 Serodiscordant Couples

**DOI:** 10.3389/fimmu.2022.813412

**Published:** 2022-03-24

**Authors:** Melinda S. Suchard, Neil Martinson, Susan Malfeld, Debbie de Assis Rosa, Romel D. Mackelprang, Jairam Lingappa, Xuanlin Hou, Helen Rees, Sinead Delany-Moretlwe, Hadassa Goldfein, Heena Ranchod, David Coetzee, Kennedy Otwombe, Lynn Morris, Caroline T. Tiemessen, Dana M. Savulescu

**Affiliations:** ^1^ National Institute for Communicable Diseases, A Division of the National Health Laboratory Service, Johannesburg, South Africa; ^2^ Chemical Pathology, School of Pathology, Faculty of Health Sciences, University of the Witwatersrand, Johannesburg, South Africa; ^3^ Perinatal Health Research Unit (PHRU), University of The Witwatersrand, Johannesburg, South Africa; ^4^ Johns Hopkins University Centre for TB Research, Baltimore, MD, United States; ^5^ School of Molecular and Cell Biology, Faculty of Science, University of the Witwatersrand, Johannesburg, South Africa; ^6^ Department of Global Health, University of Washington, Seattle, WA, United States; ^7^ Department of Obstetrics and Gynecology, University of Washington, Seattle, WA, United States; ^8^ Department of Medicine and Department of Paediatrics, University of Washington, Seattle, WA, United States; ^9^ Wits Reproductive Health and HIV Institute, Faculty of Health Sciences, University of Witwatersrand, Johannesburg, South Africa; ^10^ Division of Public Health Medicine, School of Public Health and Family Medicine, University of Cape Town, Johannesburg, South Africa; ^11^ Epidemiology and Biostatistics Department, School of Public Health, Faculty of Health Sciences, University of the Witwatersrand, Johannesburg, South Africa; ^12^ Virology Department, School of Pathology, Faculty of Health Sciences, University of the Witwatersrand, Johannesburg, South Africa

**Keywords:** HLA, heterologous, non-specific, highly exposed persistently seronegative, MHC, antibody

## Abstract

Enveloped viruses, including the Human Immunodeficiency Virus-1 (HIV), incorporate host proteins such as human leucocyte antigens (HLA) into their envelope. Pre-existing antibodies against HLA, termed HLA antibodies, may bind to these surface proteins and reduce viral infectivity. Related evidence includes macaque studies which suggest that xenoimmunization with HLA antigens may protect against simian immunodeficiency virus infection. Since HIV gp120 shows homology with class 2 HLA, including shared affinity for binding to CD4, class 2 HLA antibodies may influence HIV acquisition *via* binding to gp120 on the viral envelope. We conducted a nested case-control study on HIV serodiscordant couples, comparing the frequency of HLA antibodies among highly exposed persistently seronegative controls with those who went on to acquire HIV (HIV-seroconverters). We first performed low resolution HLA typing on 143 individuals who were HIV-infected at enrollment (index partners) and their corresponding sexual partners (115 highly exposed persistently seronegative individuals and 28 HIV-seroconverters). We then measured HLA class 1 and 2 antibodies in the highly exposed persistently seronegative individuals and HIV-seroconverters at early and late timepoints. We analyzed whether such antibodies were directed at HLA specificities of their HIV-infected index partners, and whether autoantibodies or complement-fixing class 2 HLA antibodies were present. Seventy-nine percent of highly exposed persistently seronegative individuals had HLA antibodies; 56% against class 1 and 50% against class 2 alleles. Half of the group of highly exposed persistently seronegative individuals, prior to seroconversion, expressed class 2 HLA antibodies, compared with only 29% of controls (p=0.05). HIV infection was a sensitizing event leading to *de novo* development of antibodies against HLA-A and HLA-B loci, but not against class 2 loci. HLA autoantibodies were present in 27% of highly exposed persistently seronegative individuals. Complement-fixing class 2 HLA antibodies did not differ significantly between highly exposed persistently seronegative individuals and seroconverters. In multivariable regression, presence of class 2 HLA antibodies at early timepoints was associated with reduced odds of HIV acquisition (odds ratio 0.330, confidence interval 0.112-0.976, p=0.045). These epidemiological data suggest that pre-existing class 2 HLA antibodies were associated with reduced odds of HIV acquisition.

## Introduction

Enveloped viruses derive their envelope from the host cell membrane, incorporating host proteins within the viral envelope. Such host proteins include polymorphic variants such as the human major histocompatibility antigens (MHC) called human leucocyte antigens (HLA) (1–5). The presence of antibodies against HLA could therefore impact susceptibility to natural infection with enveloped viruses. Alloimmunity is immunity of an individual to other individuals of the same species, often mediated *via* antibodies against HLA specificities (HLA antibodies), while xenoimmunity is immunity against a different species. Despite intense interest in allo- and xenoimmunity for HIV vaccine development in the early 2000’s (6) there remains no coordinated effort to explore whether intentional induction of HLA antibodies may indeed reduce HIV acquisition.

Cynomolgus macaque vaccine SIV challenge studies done in the laboratory of Dr James Stotts initially showed protection from challenge associated with an SIV vaccine and challenge virus grown in human cell lines. They determined that the correlate of protection was antibody against the cell line, specifically human MHC and not HIV-specific immune responses that were responsible for protection (7, 8). This was a model of xenoimmunization, whereby macaques were immunized by human cells. Immunizing macaques *with purified HLA-DR protein alone* could protect against subsequent SIV challenge with SIV grown in human, but not macaque, cells (9). The authors suggested that immunization with HLA-DR induced sterilizing immunity to SIV by preventing the first infectious particle from entering macaque cells, and suggested incorporation of HLA-DR into future HIV vaccines (9). Additional relevant examples include immunization of macaques with recombinant HLA class 1 or 2 proteins, either alone or in combination with HIV and SIV proteins and adjuvant (10). Complete or partial protection was achieved in macaques vaccinated with all three components and was transferrable by serum. The macaques developed anti-HLA antibodies which correlated inversely with viral load after challenge, as well as complement-dependent neutralizing activity. The authors did not identify any protection using immunization with either SHIV alone or HLA proteins alone, although there was a trend to delayed viremia in the HLA-immunized group (10).

Published work investigating the phenomenon of human HLA incorporation into the viral envelope has shown selective incorporation of HLA class 1 and 2 proteins (11). Expression of HLA-C, a class 1 HLA protein, in the viral envelope increases infectivity of virions and affects sensitivity to neutralization (12, 13). HLA-DR, a class 2 HLA protein, is incorporated into viral envelope in approximately one fifth the concentration of HIV gag protein, although HLA-DP and HLA-DQ are reportedly not incorporated (4, 14). HLA-DR in the viral envelope has been shown to be intact and functional and could stimulate T cells *via* superantigen presentation (15).

Aside from human HLA incorporation into viral membrane, HIV gp120 protein has sequence homology with class 2 HLA proteins (summarized in **Supplementary Table 1**). Both proteins bind to the CD4 receptor *in vivo*, suggesting structural homology. There are thus multiple conceptual reasons as well as suggestive animal studies that support the idea that pre-existing antibody to HLA proteins may affect HIV acquisition risk.

There has been limited epidemiological investigation exploring whether HIV-susceptible individuals prior to HIV seroconversion have pre-existing HLA antibodies that influence HIV acquisition risk. It is clear that pre-existing HLA antibodies occur in healthy individuals. Multiparous women often have detectable HLA class 1 or 2 antibodies as pregnancy is a well-described sensitizing event for their development (16–19). Newer solid phase immunoassays, with improved sensitivity over older complement dependent cytotoxicity (CDC) or enzyme-linked immunosorbent assay (ELISA) methods (16, 20, 21), have shown that individuals with no history of previous sensitizing events also harbor HLA antibodies (22–24). The natural history of HLA antibody development remains obscure and children, even neonates, can harbor HLA antibodies (25–27). Proposed mechanisms for induction of the antibodies include cross reactions with commensal flora, food antigens or allergens (22, 24, 28–30).

In highly HIV-exposed, persistently seronegative people, 30% had antibodies that reacted with gp120 and 70% had antibodies to class 1 HLA, leading the authors to hypothesize that antibodies against HLA might be protective against HIV acquisition (31). In persistently HIV seronegative intravenous drug users, antibodies against HLA (cross-reactive with HIV) were present in 33%, but in only 4% of healthy controls (32). In HIV-exposed seronegative sex workers, there was no difference in proportion with HLA class 1 antibodies between sex workers and controls; however class 2 HLA antibodies were not analyzed (33, 34).

To more conclusively associate presence of HLA antibodies with risk of HIV infection, we compared highly exposed persistently seronegative individuals to those who went on to acquire HIV (HIV-seroconverters). For this work, we have used South African samples collected during the Partners in Prevention HSV/HIV Transmission Study, hereafter termed the “Partners” study, and from the Couples Observational Study or “COS”, that prospectively followed couples serologically discordant for HIV and accurately documented HIV seroconversion. Antiretroviral therapy was consistent with national guidelines at the time of the study. We hypothesized that partner-directed HLA antibodies would be associated with lower odds of HIV acquisition. We found the presence of pre-existing class 2 HLA antibodies associated with lower odds of HIV acquisition.

## Methods

### Study Samples

The Partners study was a randomized, double blind, placebo-controlled trial recruiting HIV discordant couples to investigate the role of genital herpes simples 2 virus (HSV-2) suppression with daily acyclovir to prevent transmission of HIV from the HIV-infected partner (index partner) to their enrolled initially HIV-uninfected partner (35). The Partners study enrolled 3408 heterosexual couples from seven countries in Southern and Eastern Africa between 2004 and 2007 who had co-habited for a median of five years. and reported three or more episodes of vaginal intercourse over the prior three months (35). Enrolment criteria included age above 18 years and that the HIV infected partner (index partner) was seropositive for both HIV and HSV-2 with a CD4^+^ T cell count above 250 cells/µl. Participants were followed every three months. At every visit, couples were counselled regarding HIV risk reduction, provided with condoms, treated for any sexually transmitted infection and females tested for pregnancy. If participants met the national criteria for antiretroviral therapy at the time, they received it from local clinics. There were 132 total HIV transmissions within the 14 sites (35). The final results of the Partners study showed that acyclovir decreased the level of plasma HIV RNA in the index partner but did not reduce HIV transmission (35), thus acyclovir usage was not included in our analysis for determinants of HIV acquisition.

The COS study was conducted in South Africa and Uganda and enrolled 485 HIV serodiscordant couples. Recruitment criteria were similar to the Partners study but had no restriction based on CD4^+^ T cell count or HSV-2 infection (36). Couples were followed up quarterly for twelve months. A sampling of participants from both Partners and COS cohorts was included in an earlier genome wide association study (36) and HLA alleles associated with HIV transmission and plasma viral setpoints have been reported (37).

We accessed samples from the South African Partners and COS study sites in Johannesburg (Soweto and Orange Farm sites) and Cape Town. Originally, we included only the Soweto site of the Partners study, retrieving all samples, which comprised only 5 seroconverting couples and 68 couples who did not transmit HIV. To increase our numbers of seroconverters for meaningful statistical analysis, we requested all additional seroconverters from the Cape Town and Orange Farm sites as well as from the COS study at the Soweto site, with persistently seronegative control samples in a 2:1 ratio. Our enrollment sites are summarized in **Supplementary Figure 1** and **Supplementary Table 2**. In total there were 28 couples who experienced a HIV-seroconversion event across these three sites, which were all included in our analysis. In addition, 115 non-transmitting couples with a highly HIV-exposed, persistently seronegative partner, hereafter referred to as a “control”, were included. Samples were analyzed for HLA antibodies at two time points for each participant who was HIV-uninfected at enrolment. The first time point was at 0-6 months from enrolment and in HIV-seroconverters was prior to HIV acquisition. The second timepoint was at least 6 months from enrollment and in HIV-seroconverters was selected to be after seroconversion, ranging up to 31 months from the first timepoint.

### Linked *Versus* Unlinked Seroconversions

For both the Partners and COS studies, sequencing of a limited region of HIV *gag* and *env* genes had been performed in couples with HIV transmission, in order to determine whether the transmission was from the enrolled partner (linked) or a non-enrolled partner (unlinked) (35, 38). In our subset of South African samples from the Partners and COS studies, two thirds (19/28; 68%) of seroconversions were linked.

### Ethics

The Partners and COS studies was approved by institutional review boards at each site in each country. Participants gave informed consent, including consent for genetic testing and future research on factors associated with HIV acquisition risk. New approval for this nested case-control study was granted by the University of the Witwatersrand Human Research Ethics Committee (M1411118).

### HLA Antibody Testing and Reporting

To degrade complement, sera from HIV-seroconverters and highly exposed persistently seronegative individuals were heat inactivated at 56°C for 30 minutes after which expression levels of antibodies against HLA proteins were measured using Lifecodes single antigen LSAI and LSAII kits (Immucor, USA) containing one recombinant HLA specificity per bead. The beads were incubated with patient sera, allowing specific antibody to bind to the bead. Unbound antibody was removed through washing steps, and then bound antibody was detected by addition of phycoerythrin labelled anti-human IgG. Bead fluorescence was detected using a Luminex instrument (LX-200, Millipore, Milliplex, Germany). Highly reactive sera samples were purified by adsorption of non-specific antibodies using commercial “Sera clean” kit (Immucor, USA), followed by a second analysis. Data was imported into Match-It software (Immucor, USA) for analysis. We used manufacturer default settings for assignment of positive, negative, or weak antibodies, and manual review to confirm the instrument assignments.

Presence or absence of antibody (all antibodies or matching antibodies only) at each locus was recorded in a binary fashion in a spreadsheet (Excel, Microsoft). The number of individuals with and without antibodies were then calculated for each locus. For HLA A, B, C, DRB-1, and DRB3/4/5 loci we also reported the number of antibody specificities per individual at the four-digit resolution (for example, if an individual had antibodies against HLA-A*02:01, HLA-A*02:02 and HLA-B*27:01, the individual was considered to have a total of three class 1 antibody specificities). We could not perform this analysis for the other class 2 loci where even single antigen kits comprise more than one specificity per bead.

For analysis of complement-fixing antibodies in HIV-seroconverters and highly exposed persistently seronegative individuals, we utilized the Lifecodes C3d detection kit (ImmuCor, USA). The principle of the assay is that patient serum is allowed to bind with the HLA-coated beads as in the class 2 procedure outlined above without heat inactivation. Following incubation, negative serum reagent is added as a source of human complement, which can bind to bead-bound antibody, including that of IgG, IgM or other isotypes. The sensitized beads are washed to remove unbound antibody. An anti-human complement component C3d antibody conjugated to phycoerythrin is used as a fluorescent detection reagent.

### DNA Extraction and HLA Typing From Dried Blood Spot

DNA was extracted from dried blood spots (DBS), collected from index participants, HIV-seroconverters and highly exposed persistently seronegative individuals at enrolment. The extraction was performed using a commercial kit (Gentra^®^ Puregene^®^ Tissue Kit, Qiagen, Germany) according to instructions. The extracted DNA was quantified using a Nanodrop spectrophotometer (Thermo Fisher Scientific, USA). Commercial single stranded oligonucleotide probe kits (Lifecodes, Immucor, USA) were used for typing the following HLA loci: HLA-A, HLA-B, HLA-C, HLA-DRB1, HLA-DRB3,4,5, HLA-DQB1 and HLA-DPA/B. All kits allow for allele specific PCR amplification. Oligonucleotide probes in the kits are attached to microspheres detectable by Luminex instrument (LX-200, Milliplex, Merck), Germany. The data obtained from the typing experiments was analyzed using Match-It DNA software versions 3.18-3.24 (GenProbe, USA). The typing was reported at the two-digit resolution.

### Statistics

Demographic data and the difference in proportion of individuals with and without antibodies between the HIV-seroconverters and highly exposed persistently seronegative individuals was assessed using Fisher’s exact test. Each locus was assessed independently. No adjustments were made for multiple comparisons, rather analysis of each locus, and class 1 and class 2 antibodies overall, was conducted in an exploratory fashion to determine which variables to include further in univariate and multivariate regression for association with HIV acquisition risk. Number of antibody specificities in HIV-seroconverters compared with highly exposed persistently seronegative individuals was compared using a Mann-Whitney test for non-parametric data. P values less than or equal to 0.05 were considered significant and p values less than 0.1 were taken to suggest a trend to significance.

For group comparisons, means and standard deviations (SD) were determined for continuous measures, whereas frequencies and their percentages were determined for categorical measures. Continuous measures were compared by HIV status of the partner using the student t-test whereas the categorical measures were compared using chi-square analysis or Fisher’s exact test as appropriate. Risk factors for HIV transmission were determined by univariate and multivariate logistic regression. Factors analyzed included gender, age, cohort, ethnicity, recruitment site, number of children at enrolment, history of unprotected sex during the study (more detail below) injectable contraception, laboratory-confirmed sexually transmitted infection at enrollment (C*hlamydia trachomatis*, *Trichomonas vaginalis* or *Neisseria ghonorrhoeae*), genital ulcer disease on history or examination and viral load (earliest recorded, maximum recorded and median over study period). Three variables related to unprotected sex **–** the variables “number unprotected sex” and “proportion unprotected sex” referred to the number *of visits* and proportio*n of visits* in which the participant reported unprotected sex in the month prior to the visit, while “no condom sex” refers to the number *of unprotected sex acts* in the prior month. Regarding the dry sex, only one individual reported the practice, we therefore did not include the variable further in our analysis.

In the multivariate regression model, we applied a two-step process by first running a full multivariate (including all variables) model using the elastic net selection method that bridges the least absolute shrinkage and selection (LASSO) and ridge regression approaches. This approach identifies potential variables for consideration in the multivariate logistic regression model. The second step involved re-modelling the variables identified in step 1 to arrive at the final multivariate results. Model fit was assessed by the Hosmer-Lemeshow goodness of fit statistics. Regression results are presented as odds ratios (ORs) together with their 95% confidence intervals (95% CI). Statistical analysis was conducted in SAS Enterprise Guide 7.15 using the procedures PROC GLMSELECT and LOGISTIC (SAS Institute Inc., Cary, NC, USA).

## Results

### Gender and Study Site of Cases and Controls

We analyzed 143 index partners, who were HIV-infected at enrollment, and their corresponding sexual partners, comprising 28 HIV-seroconverters who were HIV-uninfected at enrolment but acquired HIV during their follow-up and 115 highly exposed persistently seronegative controls who remained HIV uninfected throughout follow-up (286 individuals in total). Of the 143 HIV-infected index partners, 73% of index partners to highly exposed persistently seronegative controls and 75% of index partners to HIV-seroconverters were female. Amongst couples identified with linked HIV-seroconversion (19 couples, 38 individuals), 74% of HIV-infected index partners were female. In couples where the HIV-uninfected partner at enrolment remained highly exposed persistently seronegative, the median time interval between first and second timepoints was 15 months (range 6-24 months), which was not different from the time interval between first and second timepoints in couples with a HIV-seroconversion event (median 14.5 months, range 6-31 months, p=0.900). In HIV-seroconverters, the second timepoint was a median of 9.5 months (range 2-19 months) following HIV acquisition.

Of control couples, 64.3% were from the Soweto site (59.1% from the Partners and 5.2% from COS) and of seroconverting couples 28.6% were from Soweto (17.9 from Partners and 10.7% from COS). Study site at enrolment is shown in **Supplementary Table 2**.

### Presence of HLA Antibodies at the First Timepoint

HLA antibodies in each individual participant ranged from absent to having one or multiple specificities at one or more loci. A sample of patient-level data is shown in **Supplementary Tables 3** and **4**.

**Figure 1:**
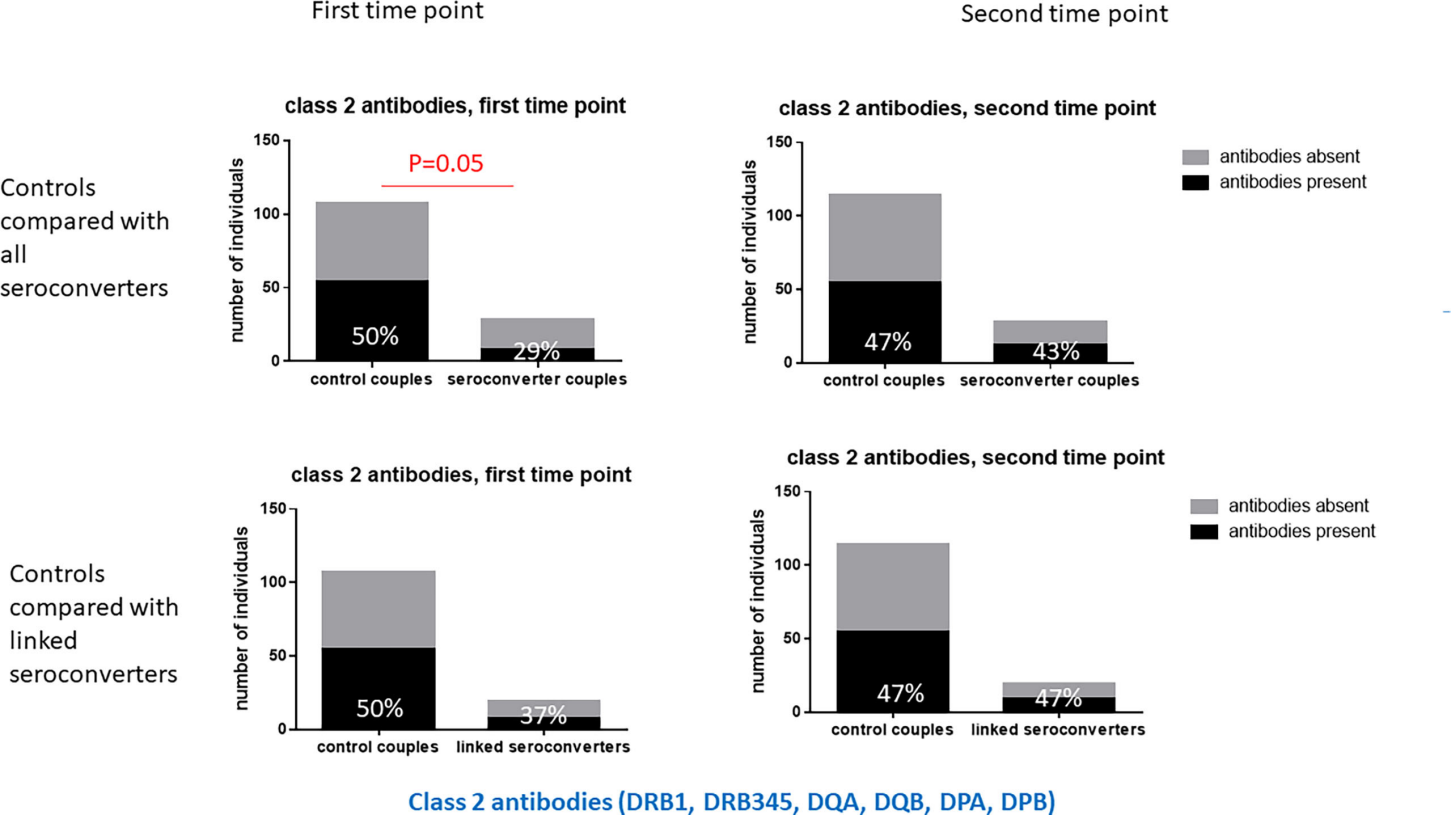
Class 2 HLA antibodies in HIV-seroconverters compared with highly exposed persistently seronegative controls, at the first and second timepoints. Upper panel shows comparison of highly expos ed persistently seronegative controls with *all* HIV-seroconverter couples. Lower panel shows comparison of highly exposed persistently seronegative controls with only *linked* HIV-seroconverter couples. At the first timepoint, a significantly higher proportion of highly exposed persistently seronegative controls than individuals who went on to acquire HIV expressed class 2 HLA antibodies.

In highly exposed persistently seronegative individuals, at the first timepoint, HLA antibodies were detected in 79% of highly exposed persistently seronegative individuals; 56% of highly exposed persistently seronegative individuals expressed antibodies against HLA class 1 and 50% against HLA class 2 (**Table 3**). The frequency of highly exposed persistently seronegative individuals expressing antibodies at individual class 1 or 2 loci ranged from 9% against HLA-C to 28% against HLA-A specificities (**Table 1**). At the first timepoint, prior to HIV-seroconversion, we noted a trend that fewer HIV-seroconverters than highly exposed persistently seronegative controls expressed any class 1 or 2 HLA antibodies (61% versus 79%, p=0.08, **Table 1**). Importantly, significantly fewer HIV-seroconverters than highly exposed persistently seronegative controls expressed HLA class 2 antibodies at this pre-seroconversion timepoint (29% versus 50%, p=0.05, **Table 1** and **Figure 1**). For each individual class 1 or 2 locus, there was no significant difference between proportion of HIV-seroconverters compared with highly exposed persistently seronegative controls expressing antibodies (**Table 1**).

**Table 1:** All HLA antibody specificities in highly exposed persistently seronegative controls compared with HIV-seroconverters (all seroconverters or only linked seroconverters), at first and second timepoints (n=115 highly exposed persistently seronegative controls, 28 HIV-seroconverters and 19 linked HIV-seroconverters).

ALL ANTIBODIES	Controls			Seroconverters (all)				Seroconverters (linked)			
Locus	% controls with Abs present	# individuals with Abs present	# individuals with Abs absent	% seroconverters with Abs present	# individuals with Abs present	# individuals with Abs absent	p value compared with controls	% linked seroconverters with Abs present	# individuals with Abs present	# individuals with Abs absent	p value compared with controls
**Time point 1**											
HLA-A antibodies	28%	30	76	39%	11	17	0.36	42%	8	11	0.28
HLA-B antibodies	26%	39	68	32%	9	19	0.82	37%	7	12	1.00
HLA-C antibodies	9%	10	97	4%	1	27	0.46	5%	1	18	1.00
Any HLA class 1 antibodies	56%	60	47	54%	15	13	0.83	58%	11	8	1.00
HLA-DRB1 antibodies	17%	18	89	11%	3	25	0.56	16%	3	16	1.00
HLA-DRB3/4/5 antibodies	11%	12	95	4%	1	27	0.30	5%	1	18	0.50
HLA-DQB1 antibodies	25%	27	80	11%	3	25	0.13	16%	3	16	0.56
HLA-DPA1 antibodies	12%	13	94	4%	1	27	0.30	0%	0	19	0.21
HLA-DPB1 antibodies	18%	19	88	7%	2	26	0.24	5%	1	18	0.30
HLA-DQA1 antibodies	23%	25	82	7%	2	26	**0.07**	11%	2	17	0.36
Any HLA class 2 antibodies	50%	54	53	29%	8	20	0.05*	37%	7	12	0.32
Any HLA antibodies	79%	84	23	61%	17	11	**0.08**	63%	12	7	0.15
**Time point 2**											
HLA-A antibodies	33%	37	76	57%	16	12	0.03*	63%	12	7	0.02*
HLA-B antibodies	26%	30	85	50%	14	14	0.02*	**42%**	8	11	0.17
HLA-C antibodies	6%	7	108	7%	2	26	1.00	**5%**	1	18	1.00
Any HLA class 1 antibodies	50%	58	57	75%	21	7	0.02*	79%	15	4	0.03*
HLA-DRB1 antibodies	18%	20	94	14%	4	24	0.75	21%	4	15	0.74
HLA-DRB3/4/5 antibodies	10%	11	103	14%	4	24	0.69	11%	2	17	1.00
HLA-DQB1 antibodies	24%	27	87	11%	3	25	0.20	11%	2	17	0.25
HLA-DPA1 antibodies	11%	12	102	0%	0	28	0.12	0%	0	19	0.21
HLA-DPB1 antibodies	13%	15	99	7%	2	26	**0.07**	**11%**	2	17	1.00
HLA-DQA1 antibodies	24%	27	87	11%	3	25	0.20	11%	2	17	0.25
Any HLA class 2 antibodies	47%	54	60	43%	12	16	0.83	47%	9	10	1.00
Any HLA antibodies	79%	89	24	86%	24	4	0.60	84%	16	3	1.00

The proportion of highly exposed persistently seronegative controls, HIV-seroconverters and linked HIV-seroconverters with antibodies at each specific locus are indicated as percentages. The number of positive and negative individuals analyzed at each locus are shown. p values were calculated using Fisher’s exact test and apply to within-row comparisons; Abs, antibodies; bold figures indicate trend to significance p<0.01; asterisk indicates significant p values<0.05. Vertical numbers are not cumulative as one individual may express antibodies to more than one HLA locus, and number of analyzable results differed per locus.

Repeating the analysis in linked HIV-seroconverters, after exclusion of couples in whom there had been HIV-seroconversion unlinked to the index partner, there was no significant difference in proportion of HLA antibodies overall or at any locus between linked HIV-seroconverters and highly exposed persistently seronegative controls (**Table 1**).

### Presence of HLA Antibodies at the Second Timepoint

**Figure 2:**
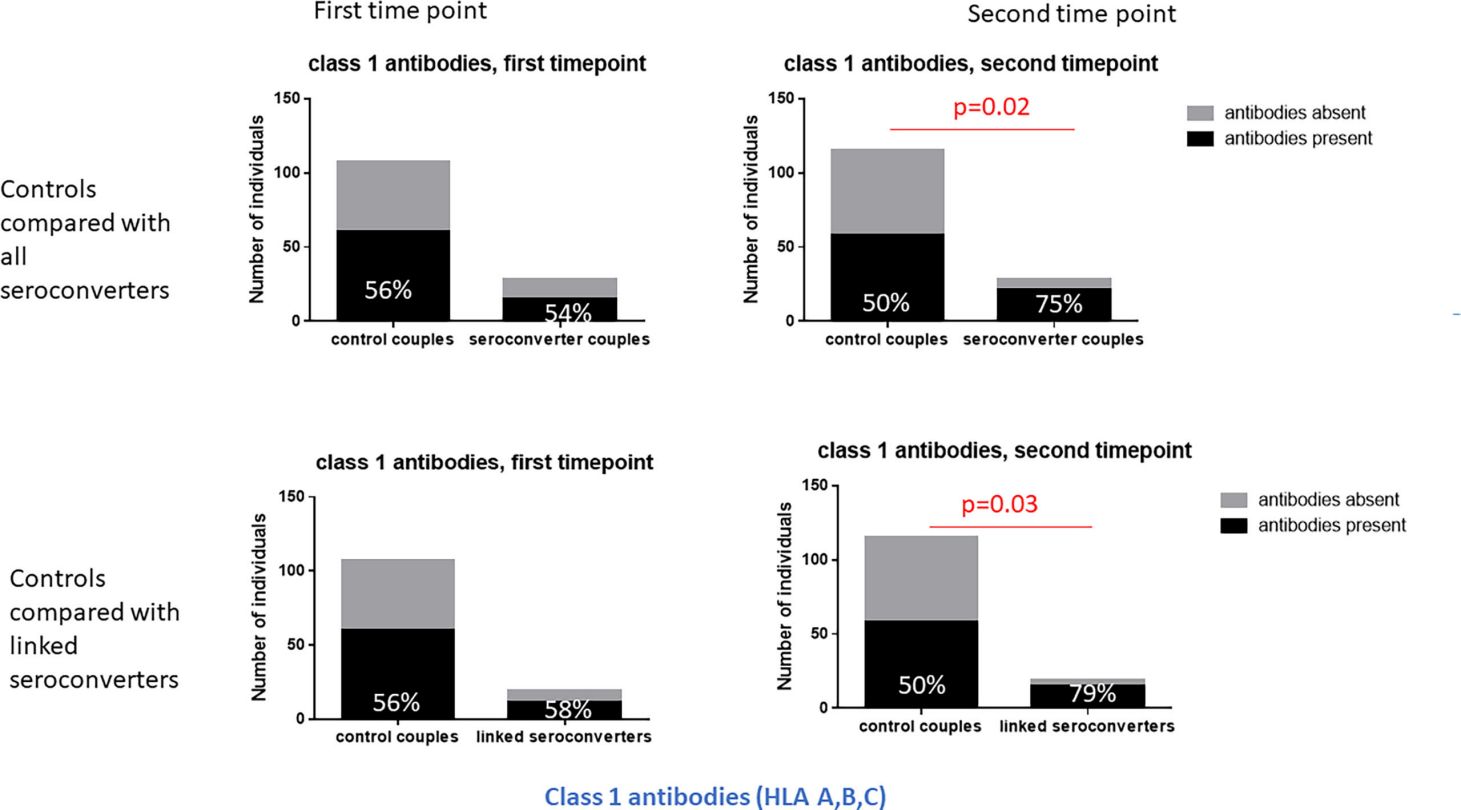
Class 1 HLA antibodies in HIV-seroconverters compared with highly exposed persistently seronegative controls, at the first and second timepoint. Upper panel shows comparison of highly exposed persistently seronegative controls with *all* HIV-seroconverter couples. Lower panel shows comparison of highly exposed persistently seronegative controls with only *linked* seroconverters. At the second timepoint, significantly more HIV-seroconverters (*all* seroconverters or *linked* seroconverters) than highly exposed persistently seronegative controls expressed class 1 HLA antibodies.

At the second timepoint, in highly exposed persistently seronegative controls, 79% of highly exposed persistently seronegative individuals expressed HLA antibodies (**Table 1**). Half of the highly exposed persistently seronegative individuals expressed antibodies against class 1 and 47% against class 2 specificities (**Table 1**). The frequency of highly exposed persistently seronegative individuals expressing antibodies at individual class 1 or 2 loci ranged from 6% for HLA-C to 33% for HLA-A (**Table 1**). In HIV-seroconverters following HIV-seroconversion, 86% of HIV-seroconverters expressed HLA antibodies - 75% against class 1 and 43% against class 2 alleles. After HIV-seroconversion, there were significantly more HIV-seroconverters than highly exposed persistently seronegative controls with class 1 HLA antibodies (75% versus 50%, p=0.02, **Table 1** and **Figure 2**). In particular, there were significantly more HIV-seroconverters than highly exposed persistently seronegative controls with antibodies against HLA-A (57% versus 33%, p=0.03, **Table 3**) and HLA-B (50% versus 26%, p=0.02, **Table 1**).

Repeating the analysis after restriction to linked HIV-seroconversions only, the same pattern was apparent. Following HIV-seroconversion, more linked HIV-seroconverters expressed class 1 antibodies than highly exposed persistently seronegative controls (79% versus 50%, p=0.03, **Table 1** and **Figure 2**). Notably there were more linked HIV-seroconverters than highly exposed persistently seronegative controls who expressed HLA-A antibodies in particular (63% versus 33%, p=0.02, **Table 1**).

### Presence of Matching Antibodies at the First Timepoint

**Figure 3:**
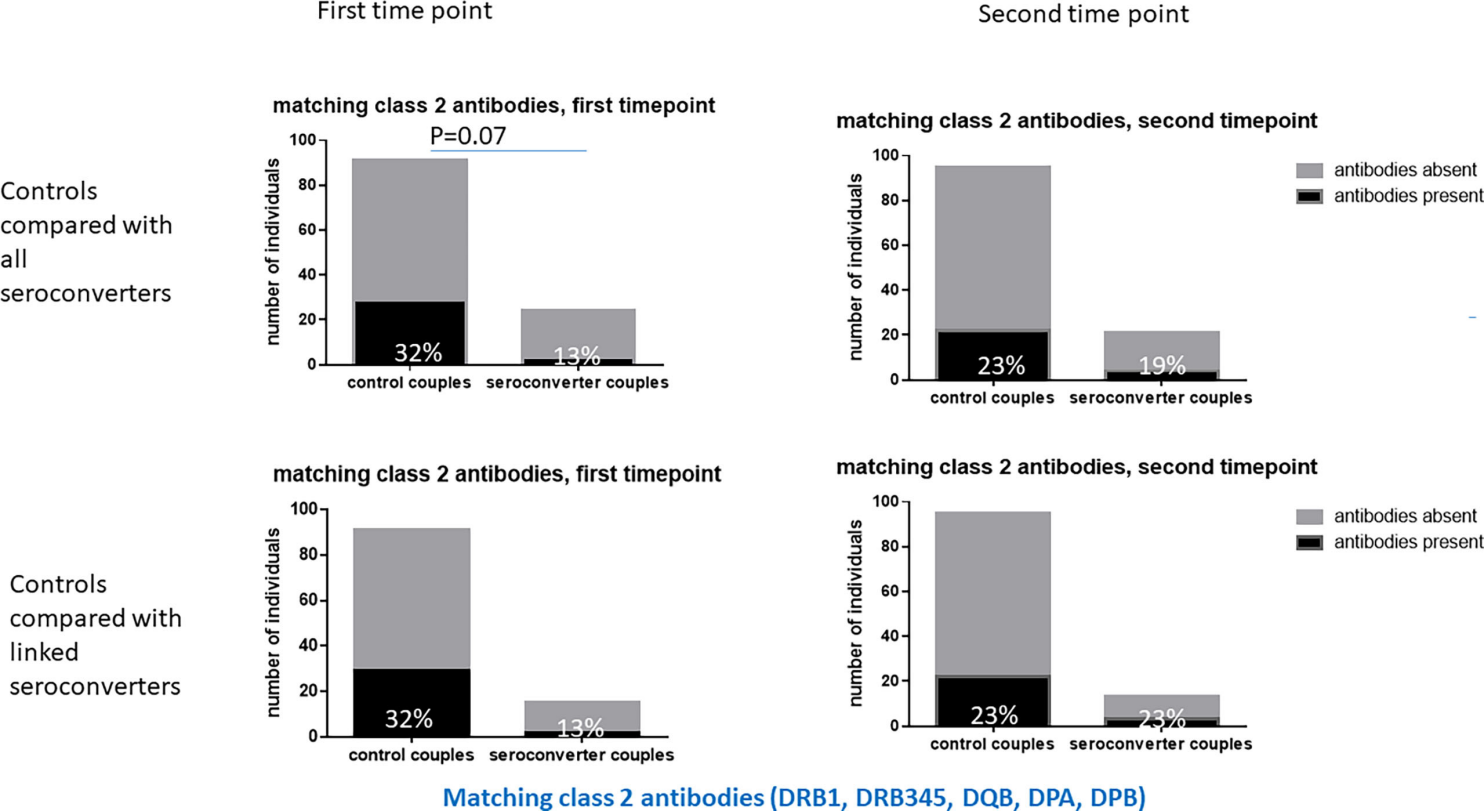
Matching class 2 HLA antibodies in HIV-seroconverters compared with highly exposed persistently seronegative controls, at the first and second timepoints. Upper panel shows comparison of highly exposed persistently seronegative controls with *all* HIV-seroconverter couples. Lower panel shows comparison of highly exposed persistently seronegative controls with only *linked* HIV-seroconverter couples. At the first timepoint there was a trend to more matching class 2 HLA antibodies in highly exposed persistently seronegative controls than in those who went on to acquire HIV.

At the first timepoint, 45% of highly exposed persistently seronegative individuals expressed HLA antibodies matching their HIV-infected index partner’s HLA type, hereafter referred to as “matching antibodies”. (**Table 2**). This figure included 13% of highly exposed persistently seronegative controls who expressed matching HLA class 1 antibodies and 32% who expressed matching HLA class 2 antibodies against their index partner’s HLA type. At individual loci, the frequency of highly exposed persistently seronegative controls with class 1 or class 2 antibodies matching their index partner ranged from 1% for HLA-C to 13% for HLA-DQB (**Table 2**). Prior to HIV-seroconversion, 30% of HIV-seroconverters expressed matching HLA antibodies of either class to their index partner’s HLA type, not significantly different from highly exposed persistently seronegative controls. 23% of HIV-seroconverters expressed matching HLA-class 1 antibodies and 13% expressed matching HLA class 2 antibodies against their index partners HLA type (**Table 2**). There was a trend to fewer HIV-seroconverters than highly exposed persistently seronegative controls expressing matching HLA class 2 antibodies against their index partner’s HLA type at this pre-seroconversion timepoint (13% versus 32%, p=0.07, **Table 2** and **Figure 3**). In HIV-seroconverters, the frequency of matching antibodies at each class 1 or 2 locus ranged from 0% (HLA C, HLA-DRB3/4/5 and HLA-DPB1) to 18% for HLA-A (**Table 2**).

**Table 2:** HLA antibodies which matched the HLA genotype of the HIV-infected index partner (“matching antibodies”) in highly exposed persistently seronegative controls compared with HIV-seroconverters (all seroconverters or linked seroconverters) at first and second timepoints (n=115 highly exposed persistently seronegative controls, 28 HIV-seroconverters and 19 linked HIV-seroconverters).

MATCHING ANTIBODIES ONLY	Controls			Seroconverters (all)				Seroconverters (linked)			
Locus	% controls with Abs	Abs present	Abs absent	% seroconverters with Abs	Abs present	Abs absent	p value compared with controls	% linked seroconverters with Abs	Abs present	Abs absent	p value compared with controls
**Time point 1**											
Matching HLA-A antibodies	5%	5	90	18%	4	22	0.10	18%	3	14	0.10
Matching HLA-B antibodies	8%	7	83	8%	2	24	1.00	12%	2	15	0.63
Matching HLA-C antibodies	1%	1	97	0%	0	27	1.00	0%	0	18	1.00
Matching HLA class 1 antibodies	13%	10	70	23%	6	20	0.21	29%	5	12	0.13
Matching HLA-DRB1 antibodies	8%	8	97	4%	1	25	0.68	6%	1	16	1.00
Matching HLA-DRB3/4/5 antibodies	8%	8	98	0%	0	27	0.36	0%	0	18	0.60
Matching HLA-DQB1 antibodies	13%	13	86	4%	1	25	0.30	6%	1	16	0.69
Matching HLA-DPA1 antibodies	8%	9	97	4%	1	27	0.69	0%	0	19	0.35
Matching HLA-DPB1 antibodies	1%	1	94	0%	0	27	1.00	0%	0	18	1.00
Matching HLA class 2 antibodies	32%	29	62	13%	3	21	**0.07**	13%	2	13	0.22
Any matching HLA antibodies	45%	36	44	30%	7	16	0.24	36%	5	9	0.57
**Time point 2**											
Matching HLA-A antibodies	4%	4	94	16%	4	21	0.05*	19%	3	13	**0.06**
Matching HLA-B antibodies	7%	7	93	12%	3	23	0.43	12%	2	15	0.62
Matching HLA-C antibodies	1%	1	109	0%	0	27	1.00	0%	0	18	1.00
Matching HLA class 1 antibodies	11%	9	76	28%	7	18	0.05*	31%	5	11	0.04*
Matching HLA-DRB1 antibodies	6%	6	102	8%	2	23	0.64	13%	2	14	0.28
Matching HLA-DRB3/4/5 antibodies	4%	4	108	4%	1	26	1.00	0%	0	18	1.00
Matching HLA-DQB1 antibodies	15%	16	92	4%	1	25	0.19	6%	1	17	0.46
Matching HLA-DPA1 antibodies	3%	3	108	0%	0	28	1.00	0%	0	19	1.00
Matching HLA-DPB1 antibodies	1%	1	102	0%	0	26	1.00	0%	0	17	1.00
Matching HLA class 2 antibodies	23%	22	73	19%	4	17	0.78	23%	3	10	1.00
Any matching HLA antibodies	40%	32	48	48%	10	11	0.62	54%	7	6	0.38

The proportion of highly exposed persistently seronegative controls, HIV-seroconverters and linked HIV-seroconverters with matching antibodies against the index partner genotype at each specific locus are indicated as percentages. The number of positive and negative individuals analyzed at each locus are also shown. p values were calculated using Fisher’s exact test and apply to within-row comparisons; Abs, Antibodies; bold figures indicate trend to significance p<0.01; asterisk indicates significant p values<0.05. Vertical numbers are not cumulative as one individual may express antibodies to more than one HLA locus, and number of analyzable results differed per locus.

Restricting analysis to seroconverters with linked HIV-transmissions only, 36% of linked HIV-seroconverters expressed matching HLA antibodies against their index partner, 29% against class 1 and 13% against class 2 specificities (**Table 2**). At each individual class 1 or 2 locus, antibodies ranged from 0% (HLA-C, HLA-DRB3/4/5, HLA-DPA1, HLA-DPB1) to 18% at HLA-A.

There was no significant difference in class 2 antibodies matching the index partner HLA type between HIV-seroconverters with linked HIV-transmissions and highly exposed persistently seronegative controls (13% versus 32%, p=0.22, **Table 2** and **Figure 3**) nor significant differences at any particular class 1 or class 2 locus.

### Matching Antibodies at the Second Timepoint

**Figure 4:**
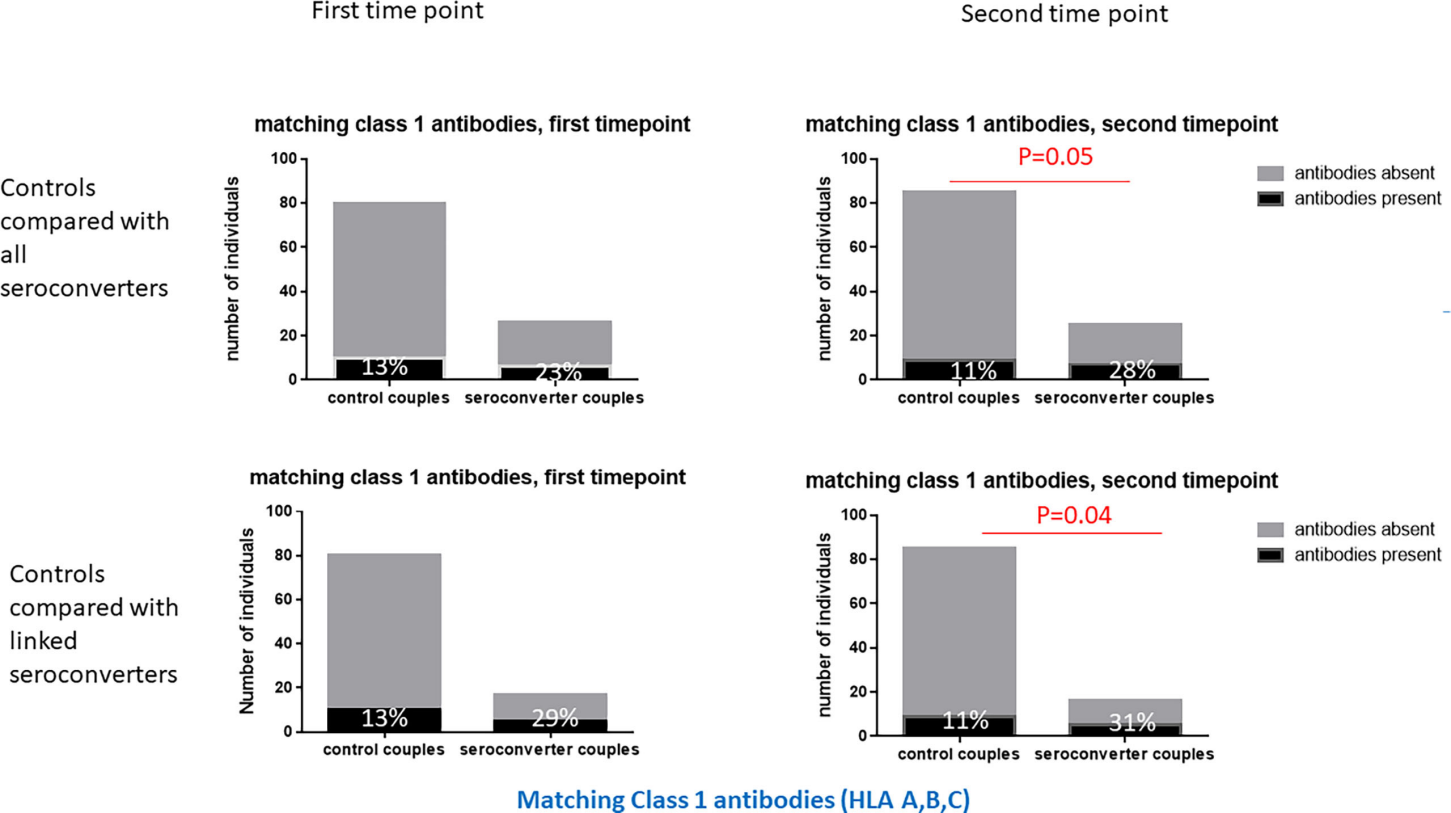
Matching class 1 HLA antibodies in HIV-seroconverters compared with highly exposed persistently seronegative controls, at the first and second timepoints. Upper panel shows comparison of highly exposed persistently seronegative controls with *all* seroconverter couples. Lower panel shows comparison of highly exposed persistently seronegative controls with only *linked* HIV-seroconverters. At the second timepoint, a significantly higher proportion of HIV-seroconverters, following HIV seroconversion, than highly exposed persistently seronegative controls expressed matching class 1 HLA antibodies.

At the second timepoint, 40% of highly exposed persistently seronegative controls expressed matching class 1 antibodies against their index partners, 11% against HLA class 1 and 23% against class 2 specificities (**Table 2**). Post HIV-seroconversion, 48% of HIV-seroconverters expressed matching HLA antibodies against their index partners, 28% against class 1 and 19% against class 2 specificities. Significantly more HIV-seroconverters, after seroconversion, expressed matching class 1 HLA antibodies than highly exposed persistently seronegative controls (28% versus 11%, p=0.05, **Table 2** and **Figure 4**), particularly at the HLA-A locus (16% versus 4%, p=0.05, **Table 2**).

Restricting analysis to linked HIV transmissions only, 54% of HIV-seroconverters expressed matching HLA antibodies against their index partners, 31% against HLA class 1 and 23% against HLA class 2 specificities. Similarly, there were significantly more linked HIV-seroconverters, post seroconversion, with class 1 HLA antibodies than highly exposed persistently seronegative controls (31% versus 11%, p=0.04, **Table 2** and **Figure 4**), with a trend to more linked seroconverters than highly exposed persistently seronegative controls with HLA-A antibodies (19% versus 4%, p=0.06, **Table 2**).

### Number of Antibody Specificities

**Figure 5:**
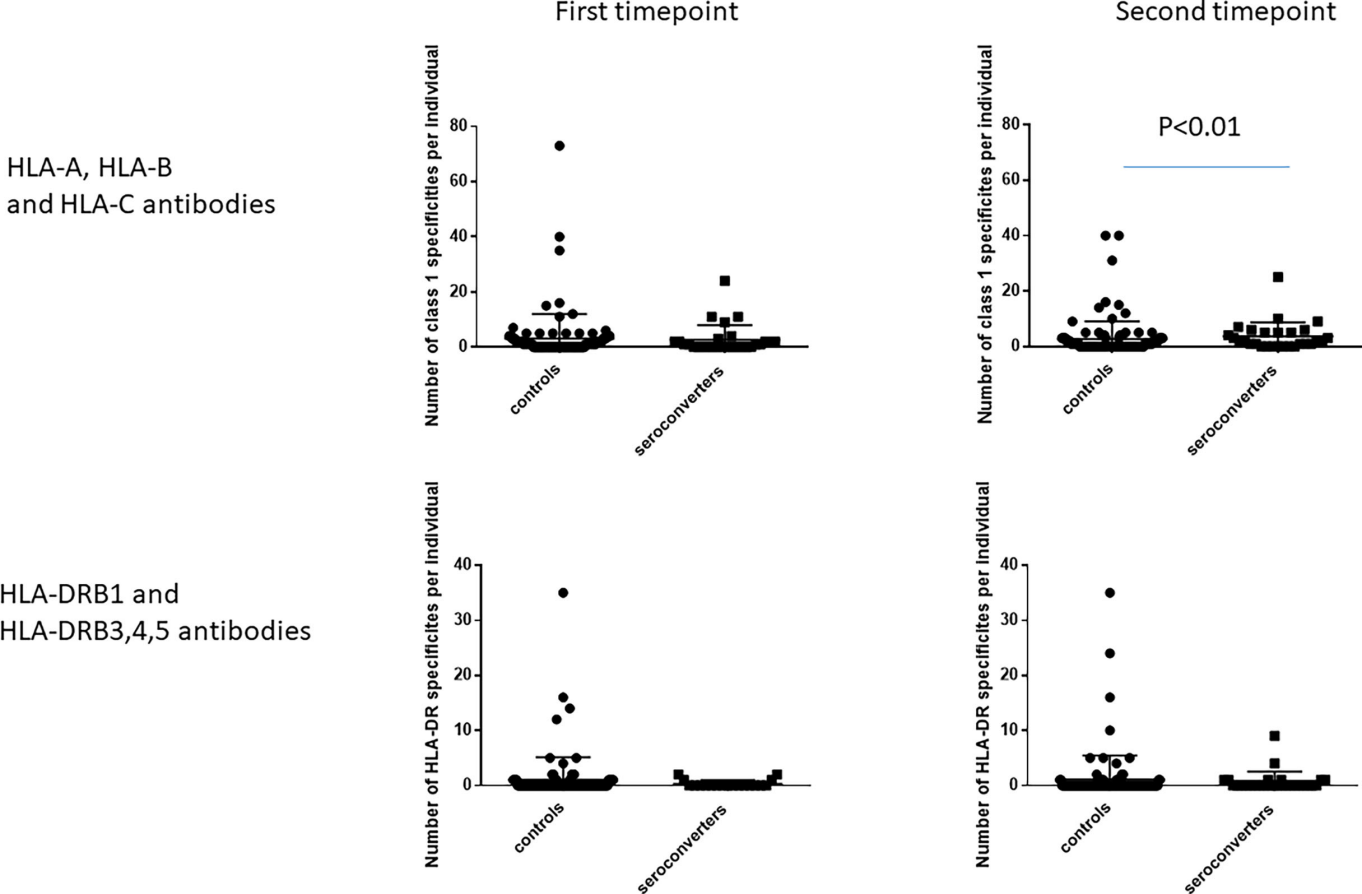
Number of HLA antibody specificities per individual, at the first and second timepoints. The left hand graphs show HLA-A, HLA-B and HLA-C antibody specificities. The right hand graphs show HLA-DRB1 and HLA-DRB3,4,5 antibody specificities. At the second timepoint, HIV-seroconverters had a median of two antibody specificities per person compared with highly exposed persistently seronegative controls who had a median of one antibody specificity per person. There was no other significant difference noted.

At the first timepoint, the number of class 1 antibody specificities per highly exposed persistently seronegative individual ranged from 0 to 73 (median 1, IQR 3), not differing significantly from HIV-seroconverters who had a range of 0-24 specificities per individual (median 1; IQR 2, **Figure 5**). For class 2 specificities, number of antibody specificities per individual ranged from 0 to 35 (median 0, IQR 0) in highly exposed persistently seronegative controls, also not differing from HIV-seroconverters, who had a range of 0 to 2 (median 0, IQR 0) specificities per individual.

At the second timepoint, the number of class 1 specificities per individual ranged from 0 to 40 (median 0, IQR 3) in highly exposed persistently seronegative controls, significantly lower than in HIV-seroconverters post seroconversion (range 0 to 25; median 2; IQR 4.75, p<0.01, **Figure 5**). For class 2, highly exposed persistently seronegative controls had 0 to 35 (median 0, IQR 0) specificities per individual, not differing from HIV-seroconverters post seroconversion who had a range of 0 to 9 (median 0, IQR 1) class 2 specificities per individual (**Figure 5**).

### Autoantibodies

HLA autoantibodies, analyzed against self HLA genotypes at two-digit resolution, were not uncommon. At the first timepoint, 27% of highly exposed persistently seronegative controls expressed autoantibodies to at least one locus, 6.7% against at least one class 1 locus and 18.2% against at least one class 2 locus. At early timepoints, there was a trend to a higher frequency of class 2 HLA autoantibodies in highly exposed persistently seronegative controls (18.2% compared with 0% of HIV-seroconverters, p=0.07, **Table 3**), in keeping with the finding of more class 2 HLA antibodies generally in highly exposed persistently seronegative controls at the early timepoint.

**Table 3:** HLA autoantibodies in highly exposed persistently negative controls compared with HIV-seroconverters, at first and second timepoints (n=115 highly exposed persistently seronegative controls, 28 HIV-seroconverters and 19 linked HIV-seroconverters).

AUTOANTIBODIES ONLY	Controls			Seroconverters			p value compared with controls
Locus	% controls with autoAbs	# individuals with autoAbs present	# individuals with autoAbs absent	% seroconverters with autoAbs	# individuals with autoAbs present	# individuals with autoAbs absent	
**Time point 1**							
HLA-A autoantibodies	2.4	2	82	0.0	0	24	1.00
HLA-B autoantibodies	3.3	3	87	8.0	2	23	0.30
HLA-C autoantibodies	0.0	0	99	0.0	0	27	1.00
Any HLA class 1 autoantibodies	6.7	5	70	8.3	2	22	0.68
HLA-DRB1 autoantibodies	5.8	6	98	0.0	0	24	0.59
HLA-DRB3/4/5 autoantibodies	2.8	3	103	0.0	0	27	1.00
HLA-DQB1 autoantibodies	7.3	7	89	0.0	0	25	0.34
HLA-DPA1 autoantibodies	1.0	1	95	0.0	0	27	1.00
HLA-DPB1 autoantibodies	0.0	0	89	0.0	0	26	1.00
HLA-DQA1 autoantibodies	0.0	0	83	0.0	0	26	1.00
Any class 2 autoantibodies	18.2	14	63	0.0	0	20	**0.07**
Any class 1 or 2 autoantibodies	27	18	42	10.5	2	17	0.13
**Time point 2**							
HLA-A autoantibodies	0.0	0	85	0.0	0	24	1.00
HLA-B autoantibodies	5.1	5	94	16.7	4	20	**0.07**
HLA-C autoantibodies	0.0	0	106	0.0	0	27	1.00
Any HLA class 1 autoantibodies	6.5	5	72	17.4	4	19	0.21
HLA-DRB1 autoantibodies	2.8	3	105	0.0	0	24	1.00
HLA-DRB3/4/5 autoantibodies	1.8	2	111	3.7	1	26	0.48
HLA-DQB1 autoantibodies	7.7	8	96	0.0	0	25	0.35
HLA-DPA1 autoantibodies	0.0	0	102	0.0	0	28	1.00
HLA-DPB1 autoantibodies	0.0	0	97	0.0	0	26	1.00
HLA-DQA1 autoantibodies	0.0	0	87	0.0	0	25	1.00
Any class 2 autoantibodies	15.6	12	65	5.3	1	18	0.45
Any class 1 or 2 autoantibodies	27.4	17	45	27.8	5	13	1.00

The proportion of highly exposed persistently seronegative controls and HIV-seroconverters with autoantibodies against their own genotype at each specific locus are indicated as percentages. The number of positive and negative individuals analyzed at each locus are also shown. p values were calculated using Fisher’s exact test and apply to within-row comparisons; Abs, antibodies; bold figures indicate trend to significance approaching p=0.05. Vertical numbers are not cumulative as one individual may express antibodies to more than one HLA locus, and number of interpretable results differed per locus.

At later timepoints, HIV-seroconverters, following seroconversion, tended to have a higher frequency than highly exposed persistently seronegative controls of HLA-B autoantibodies (16.7% in HIV-seroconverters compared with 5.1% in controls, p=0.07, **Table 3**) with no significant differences from highly exposed persistently seronegative controls in HLA autoantibody frequency at other loci.

### Complement-Fixing Antibodies

In order to expand our search to antibodies of isotypes other than IgG, we analyzed complement-fixing class 2 HLA antibodies. We considered only the first timepoint as we were particularly interested in whether complement-fixing antibodies, incorporating antibodies of IgM isoype, could differ between HIV-seroconverters and highly exposed persistently seronegative individuals, potentially conferring protection against HIV acquisition. We found that 7.5% of highly exposed persistently seronegative controls and 4.0% of HIV-seroconverters expressed complement-fixing class 2 HLA antibodies at the early timepoint. The specificities against which the complement-fixing antibodies were directed are shown in **Supplementary Tables 3** and **5**. There was no significant difference between highly exposed persistently seronegative controls and HIV-seroconverters regarding the proportion of individuals with complement-fixing class 2 HLA antibodies.

### HIV-Infected Index Partners in HIV-Transmitting and HIV-Non-Transmitting Couples

Epidemiological characteristics of HIV-infected index partners is shown in **Table 4**. The only factors differing between those who transmitted HIV to their partners from those that did not were a higher viral load (earliest viral load 4.71 versus 4.16 copies/ml, p<0.01; maximal log viral load 4.97 versus 4.55 copies/ml p=0.03) and a higher proportion who reported ever having had unprotected sexual intercourse with their partner (89% versus 56%, p<0.01). Reported number and proportion of unprotected sexual episodes did not differ between transmitting and non-transmitting individuals.

**Table 4:** Characteristics of the index partners (partners who were HIV-infected at enrolment) (n=115 index partners to highly exposed persistently seronegative controls and 19 index partners to linked seroconverters).

Characteristics of Index Partners (excluding unlinked seroconverters)			
Variable	HIV non-transmitters	HIV transmitters	p value
Mean (SD) age at enrolment (years)	33.34 (7.53)	34.00 (9.72)	0.7355
Gender n (%)			
Female	84 (73.04)	14 (73.68)	1.0000
Male	31 (26.96)	5 (26.32)	
Cohort n (%)			
Cos	6 (5.22)	3 (15.79)	0.1169
Hsv	109 (94.78)	16 (84.21)	
Ethnicity n (%)			
Other	29 (25.22)	2 (10.53)	0.4003
Sotho	17 (14.78)	2 (10.53)	
Xhosa	46 (40.00)	9 (47.37)	
Zulu	23 (20.00)	6 (31.58)	
Site n (%)			
CT	28 (24.35)	8 (42.11)	0.1595
JHB	87 (75.65)	11 (57.89)	
Mean (SD) number of children at enrolment	1.75 (1.40)	1.16 (0.96)	0.0809
Ever unprotected sex n (%)	51 (44.35)	2 (10.53)	**0.0049**
No	64 (55.65)	17 (89.47)	
Yes			
Mean (SD) Proportion unprotected sex	0.15 (0.22)	0.14 (0.13)	0.8767
Mean (SD) Number unprotected sex	2.47 (4.26)	2.05 (2.46)	0.6789
Mean (SD) No. of no condom sex acts	18.14 (56.72)	8.05 (12.84)	0.4429
Contraception injection n (%)			
0	69 (60.00)	11 (57.89)	1.0000
1	46 (40.00)	8 (42.11)	
Chlamydia n (%)			
Negative	99 (93.40)	15 (93.75)	1.0000
Positive	7 (6.60)	1 (6.25)	
Trichomonas n (%)			
Negative	79 (74.53)	11 (68.75)	0.7608
Positive	27 (25.47)	5 (31.25)	
Genital ulcers n (%)			
No	85 (73.91)	12 (63.16)	0.4064
Yes	30 (26.09)	7 (36.84)	
Any STI n (%)			
Negative	76 (71.70)	11 (68.75)	0.7744
Positive	30 (28.30)	5 (31.25)	
Mean (SD) Earliest Log VL	4.16 (0.66)	4.71 (0.73)	0.0017*
Mean (SD) Maximum Log VL	4.55 (0.76)	4.97 (0.78)	0.0294*

STI, sexually transmitted infection; VL, viral load. Statistically significant values shown in bold with asterisk.

### Highly Exposed Seronegative Partners Compared With HIV-Seroconverters

Regarding demographic and clinical parameters in partners who were HIV-uninfected at enrolment, there was no significant difference in age, gender, cohort or ethnicity between HIV-seroconverters and highly exposed persistently seronegative individuals (**Table 5**). Amongst HIV-seroconverters, 50% came from Johannesburg and 50% from Cape Town, while amongst highly exposed persistently seronegative individuals, 76.1% were recruited from Johannesburg sites, showing significant difference in proportion between cities (p=0.01) (**Table 5**).

**Table 5:** Characteristics of individuals who were HIV-uninfected at enrolment (n=115 highly exposed persistently seronegative controls and 28 HIV-seroconverters).

Characteristics of the partner who was HIV-uninfected at enrolment			
Variable	Did not acquire HIV	Acquired HIV	p value
Mean (SD) age at enrolment (years)	35.81 (9.12)	35.20 (9.94)	0.7557
Gender n (%)			
Female	31 (27.19)	7 (25.00)	1.0000
Male	83 (72.81)	21 (75.00)	
Cohort n (%)			
Cos	6 (5.26)	3 (10.71)	0.3800
Hsv	108 (94.74)	25 (89.29)	
Ethnicity n (%)			
Other	29 (25.22)	4 (14.29)	0.2985
Sotho	27 (23.48)	5 (17.86)	
Zulu	59 (51.30)	19 (67.86)	
Site n (%)			
CT	27 (23.89)	14 (50.00)	0.0100*
JHB	86 (76.11)	14 (50.00)	
Mean (SD) number of children at enrolment	1.75 (1.40)	1.32 (1.12)	0.1390
Ever unprotected sex n (%)			
No	52 (45.61)	12 (42.86)	0.8349
Yes	62 (54.39)	16 (57.14)	
Mean (SD) Proportion unprotected sex	0.20 (0.27)	0.17 (0.19)	0.6201
Mean (SD) Number unprotected sex	1.27 (1.93)	1.11 (1.29)	0.6689
Mean (SD) No. of no condom sex acts	9.77 (22.64)	6.39 (11.65)	0.4463
Contraception injection n (%)			
0	102 (89.47)	24 (85.71)	0.5210
1	12 (10.53)	4 (14.29)	
Mean (SD) Interval between 2 timepoints (mo)	14.29 (5.08)	15.46 (6.80)	0.3194
Chlamydia n (%)			
Negative	99 (93.40)	24 (96.00)	1.0000
Positive	7 (6.60)	1 (4.00)	
Trichomonas n (%)			
Negative	95 (89.62)	20 (80.00)	0.1883
Positive	11 (10.38)	5 (20.00)	
Genital ulcers n (%)			
No	85 (74.56)	21 (75.00)	1.0000
Yes	29 (25.44)	7 (25.00)	
Any STI n (%)			
Negative	93 (87.74)	19 (76.00)	0.2017
Positive	13 (12.26)	6 (24.00)	

STI, sexually transmitted infection; VL, viral load. Statistically significant values shown in bold with asterisk.

Given that pregnancy is known to be a trigger event for development of HLA antibodies, we analyzed numbers of pregnancies in the females originally HIV-uninfected at enrolment, of whom there were 31 highly exposed persistently seronegative females and 7 HIV-seroconverter females. At enrolment, the mean number of children in highly exposed persistently seronegative controls was 1.75 (SD 1.40) compared with 1.32 (SD 1.12) in HIV-seroconverters, with no significant difference between the groups (**Table 5**). Regarding pregnancies occurring during the study, pregnancy was reported by 6 of 31 highly exposed persistently seronegative females (19.4%) and 3 of 7 (42.9%) HIV-seroconverter females with no significant difference between the groups (data not shown, too few events for inclusion in regression analysis).

Regarding factors in the HIV-uninfected partner at enrollment that may be associated with HIV acquisition, the history of number of visits and proportion of visits in which unprotected sexual episodes were reported was similar in both groups (**Table 5**). Similarly the number of reported unprotected sex acts was similar in both groups as was the history of ever having had unprotected sex (57% versus 54%, **Table 5**). There was no difference in proportion of HIV-seroconverters and highly exposed persistently seronegative controls using injectable contraception. *Trichomonas vaginalis*, *Chlamydia trachomatis* and *Neisseria ghonorrhoeae* results at enrollment showed no difference between groups. Similarly, genital ulcer frequency was similar in both groups. HLA antibody variables summarized in **Table 5** were investigated in univariate and multivariate regression.

### Epidemiological Factors in Partners Who Were HIV-Uninfected at Enrolment Associated With HIV-Acquisition Risk

In univariate analysis of risk factors in the partner who was HIV-uninfected at enrolment and incorporating viral load of the HIV-infected index partner, factors significantly associated with HIV acquisition included recruitment city, index partner viral load and any class 2 HLA antibodies at the early timepoint (**Table 6**). The Cape Town site was associated with a higher odds of HIV acquisition (OR 3.185, CI 1.351-7.510, p=0.01). Class 2 HLA antibodies at the early timepoint were associated with significantly lower odds of HIV acquisition (OR 0.393, CI 0.159-0.969, P=0.04) and each log viral load in the index partner showed odds ratio of 2.192 (CI 1.137-4.225, P=0.02) for HIV acquisition by the partner who was HIV-uninfected at enrolment (**Table 6**). In multivariate analysis, the only factors significantly associated with HIV acquisition was city of recruitment (OR 2.971 CI 1.029-8.580 p=0.04) and any class 2 HLA antibodies at the first timepoint (OR 0.330 CI 0.112-0.976, p=0.045).

**Table 6:** Risk factors for HIV acquisition by individuals who were HIV-uninfected at enrolment, by univariate and multivariate regression (n=115 highly exposed persistently seronegative controls and 28 HIV-seroconverters).

HIV-seroconverters versus highly exposed persistently seronegative controls				
	Univariate		Multivariate	
Variable	OR (95% CI)	p value	OR (95% CI)	p value
Gender: Females vs Males	0.892 (0.345-2.307)	0.8144		
Enrolment age (years)	0.993 (0.949-1.039)	0.7537		
Cohort: Cos vs Partners	2.160 (0.505-9.232)	0.2988		
Ethnicity: Sotho vs Other	1.343 (0.326-5.529)	0.6833		
Xhosa vs Other	2.335 (0.727-7.494)	0.1542		
Zulu vs Other				
Site: Cape Town vs Johannesburg	3.185 (1.351-7.510)	0.0081*	2.971 (1.029-8.580)	0.0441*
Number of children at enrolment	0.765 (0.536-1.092)	0.1395	0.777 (0.497-1.215)	0.2681
Ever unprotected sex: No vs Yes	0.894 (0.388-2.060)	0.7928		
Proportion unprotected sex	0.646 (0.116-3.592)	0.6180		
Number unprotected sex	0.948 (0.743-1.210)	0.6670		
No. of no condom sex acts	0.990 (0.964-1.017)	0.4510		
Contraception injection	1.417 (0.420-4.779)	0.5742		
Interval between 2 timepoints (mo)	1.040 (0.963-1.122)	0.3178		
Chlamydia: Positive vs Negative	0.589 (0.069-5.020)	0.6285		
Trichomonas: Positive vs Negative	2.159 (0.676-6.901)	0.1942		
Genital ulcers: Yes vs No	0.977 (0.377-2.536)	0.9620		
Any STI: Positive vs Negative	2.259 (0.763-6.692)	0.1413	2.878 (0.772-10.72)	0.1152
Earliest Log VL	2.192 (1.137-4.225)	0.0191*	1.740 (0.831-3.644)	0.1416
Max Log VL	1.995 (1.119-3.557)	0.0192*		
Median Log VL	1.920 (1.058-3.484)	0.0320*		
Any class 1 HLA Abs first timepoint: yes vs no	0.904 (0.392-2.083)	0.8124		
Number of class 1 specificities first timepoint	0.993 (0.938-1.051)	0.8076		
Any class 2 HLA abs first timepoint: yes vs no	0.393 (0.159-0.969)	0.0425*	0.330 (0.112-0.976)	0.0452*
Number of DRB1 and DRB345 specificities first timepoint	0.750 (0.423-1.328)	0.3239		
Matching class 1 first timepoint: yes vs no	2.100 (0.680-6.485)	0.1972		
Matching class 2 first timepoint: yes vs no	**0.305 (0.084-1.107)**	**0.0710**		

STI, sexually transmitted infection; VL, viral load. Statistically significant values shown in bold with asterisk.

We repeated univariate and multivariate regressions on the partner HIV-uninfected at enrolment, split by gender (**Supplementary Table 6** for males and **Supplementary Table 7** for females). For males, in univariate analysis, city of recruitment, maximum viral load and matching class 1 antibodies at the first timepoint were significantly associated with increased odds of HIV acquisition. Matching class 1 HLA antibodies at the early timepoint gave an odds ratio of 6.333 (CI 1.357-29.55, P=0.02) for HIV acquisition. Interpretation is limited by the low numbers (84 male highly exposed persistently seronegative controls, 21 male HIV-seroconverters) and resultant wide confidence interval. Any class 2 HLA antibodies at the first timepoint showed a protective trend (OR 0.346 CI 0.115-1.038, p=0.06), as did matching class 2 antibodies at the early timepoint (OR 0.149 CI 0.018-1.196, p=0.07), earliest viral load and any STI (**Supplementary Table 6**). The only variable significant in multivariate analysis for male partners who were HIV-uninfected at enrolment was city of recruitment (**Supplementary Table 6**).

In female partners, HIV-uninfected at enrolment, analyzed alone (31 female highly exposed persistently seronegative controls and 7 female HIV-seroconverters), no variables were significantly associated with odds of HIV acquisition in univariate analysis, likely due to low sample numbers, therefore multivariable analysis was not performed.

### Epidemiological Factors in Index Partners Who Were HIV-Infected at Enrolment Associated With HIV-Transmission Risk

Factors in index partners who were HIV-infected at enrolment that may be associated with transmission risk were analyzed in univariate and multivariate regression (**Supplementary Table 8**). Regarding pregnancy in HIV-infected index partners, there were 11 pregnancies in 84 female index partners to highly exposed persistently seronegative controls and 2 pregnancies in 21 female index partners to HIV-seroconverters, yielding no difference between the groups and too few events for inclusion in regression analysis. For the regression analysis, we excluded unlinked couples as their index partners did not transmit HIV. In univariate regression, factors associated with increased transmission from HIV-infected index partners included reported unprotected sex and viral load measurements. There was a trend to reduced HIV transmission with increased number of children at enrollment in the HIV-infected index partner (OR 0.663 CI 0.418-1.051, p=0.08), but no association with enrollment age. In multivariate analysis, median viral load and history of unprotect ted sex remained significant and there was a trend to reduced transmission with increased number of children at enrollment in the HIV-infected index partner (OR 0.576 CI 0.329-1.007, p=0.05).

We then stratified HIV-infected index partners by gender (**Supplementary Table 9** for males and **Supplementary Table 10** for females). In males, no variable was significant on univariate regression, with only median viral load showing a trend to increased transmission risk. In females, in univariate analysis, history of unprotected sex and viral load indicators were associated with increased transmission risk. In multivariate analysis, higher number of children at enrollment in female HIV-infected index partners showed a trend to reduced odds of HIV transmission to their sexual partner (OR 0.558, CI 0.302-1.030, p=0.06).

## Discussion

To explore the role of HLA antibodies in risk of HIV acquisition, we retrospectively studied samples from the Partners and COS studies, longitudinal analyses of HIV serodiscordant couples with both genetic material and sera available for study, including timepoints prior to HIV seroconversion. The cohorts were well characterized, including viral analysis of the seroconverting couples which could identify those who acquired HIV from their index partner (linked transmissions) and those who acquired HIV from a third party (unlinked transmissions). The HIV-uninfected partners at enrolment who did not seroconvert represent healthy individuals with no known comorbidities and can be considered highly exposed persistently seronegative individuals, as most reported episodes of unprotected sexual intercourse despite HIV risk counselling (**Table 1**). We analyzed the presence of any HLA antibody at each locus in HIV-seroconverters and highly exposed persistently seronegative controls to ascertain whether particular specificities may play a prominent role. We then analyzed a subset of HLA antibodies, namely partner-directed HLA antibodies (“matching antibodies”), which could potentially react with the HIV donor’s HLA proteins. We performed these analyses twice – firstly incorporating all HIV-seroconverters, to look for general factors in persistently seronegative individuals which may differ from individuals who acquired HIV. Secondly, we repeated the analysis using only linked seroconverters, to look for associations directly related to interaction between the sexual partners between whom HIV was transmitted.

At the first timepoint, prior to seroconversion, we found that significantly fewer HIV-seroconverters had class 2 HLA antibodies compared to highly exposed persistently seronegative controls. There was no particular class 2 locus in which the proportion of highly exposed persistently seronegative controls and HIV-seroconverters differed significantly, but for each class 2 locus there were fewer HIV-seroconverters than highly exposed persistently seronegative controls with antibodies. These findings suggest that class 2 HLA antibodies are protective against HIV acquisition.

We went further to analyze the matching antibodies directed particularly against HLA types of the index partner who was HIV-infected at enrolment. Prior to HIV-seroconversion there was a similar trend noted of fewer matching HLA class 2 antibodies in HIV-seroconverters than in highly exposed persistently seronegative controls. Thus, prior to HIV-seroconversion, HIV-seroconverters showed less alloimmunity against their partners than the highly exposed seronegative control group.

There are two schemas for interaction of HLA antibodies with HIV. The first is relevant to any enveloped virus. Enveloped viruses, such as HIV and measles, bud from the host cell membrane and incorporate host proteins, including HLA, into the envelope (14, 39, 40). HLA proteins are present in the HIV envelope in higher quantities than gp120 trimers (41, 42). Incorporation of host HLA within the viral envelope of an HIV “donor” suggests that antibodies against HLA in the HIV “recipient” may play a role in protection from acquisition of HIV, similar to the role of HLA antibodies in humoral rejection of a solid organ transplant.

The second schema, of relevance only for HIV, is that HIV proteins, in particular gp120, show particular homology to HLA proteins, likely due to shared affinity for binding to the host CD4 protein. Notable homologies between HIV and host proteins has been summarized in **Supplementary Table 1** (31, 43–51) including additional homologies reported between class 2 HLA and other HIV proteins including gp41 and Nef (45, 52–54). HIV gp120 has even been shown to be able to present peptide as antigen, analogous to peptide presentation by class 2 HLA (41, 55). Some authors have speculated that HIV gp120 may have evolved from class 2 HLA or by mimicry of class 2 HLA (53, 56).

Due to homologies between HIV proteins and HLA proteins, antibodies reactive against HLA may cross-react with gp120 or other HIV antigens as reported by others using *in vitro* models (57). HLA-DR antiserum interfered with HIV replication in culture (58) and in some cases HLA class 1 and class 2 antibodies were able to neutralize strains of HIV (59). Other antibody-mediated functions such as antibody-dependent cellular cytotoxicity or antibody-dependent cell mediated viral inhibition may play a role *in vivo* (60). Complement fixation has been shown to be important in HIV transmission (1), however we found no difference between highly exposed persistently seronegative controls and HIV-seroconverters in proportion expressing complement-fixing antibodies against class 2 HLA. Alternatively, HLA antibodies may be a surrogate or correlate of protective T cell or innate immune responses.

Interestingly, while we found statistical difference between highly exposed persistently seronegative controls and HIV-seroconverters when including all HIV-seroconverters, we did not note any difference in class 2 HLA antibody frequencies comparing only linked HIV-seroconverters with highly exposed persistently seronegative controls. Thus, presence of class 2 HLA antibodies seemed protective, even against HIV acquisition from a third partner who was not enrolled in the trial. While lack of significant associations in the linked group may be an artefact of small numbers of linked transmissions, these findings may also infer that protection derived from HLA class 2 antibodies is directed at HIV gp120 itself, which would apply to HIV acquisition from any partner, rather than specifically directed towards donor HLA proteins incorporated into the viral envelope which would apply only between linked partners. That is, the protection afforded by class 2 HLA antibodies does not seem to derive from matching antibodies directed at the HIV-infected index partner’s HLA type only, but rather from any class 2 antibodies. However, donor-directed antibodies (matching antibodies) also showed a trend towards expression in more highly exposed persistently seronegative controls than HIV-seroconverters (32% versus 17%, p=0.07). While such partner-directed antibodies may be of relevance in binding to partner HLA incorporated into viral envelope, they may be a result of stimulation by exposure to the partner which then play a role in binding to HIV gp120 regardless of partner HLA incorporation into the viral envelope.

There are important implications from our work, including absence of certain findings. A relevant finding was the lack of any one particular HLA antibody allele or locus specificity common to the majority of highly exposed persistently seronegative controls. Further, we did *not* find that *only* partner-directed antibodies were associated with lower odds of HIV acquisition. Similarly, we did not demonstrate any protection from complement-fixing antibodies against class 2 HLA, thus there is no suggestion that it is antibodies of IgM isotype or newly developed antibodies that explain any protective effect.

Using multivariable regression to control for well described risk factors for HIV acquisition, in partners originally HIV-uninfected at enrolment, presence of any class 2 HLA antibodies at the early timepoint were significantly associated with lower odds of HIV acquisition in both univariate (OR 0.393 CI 0.159-0.969, p=0.04) and multivariate analysis (OR 0.330, CI 0.112-0.976, P=0.045). This association was stronger for originally HIV-uninfected partners than traditional risk factors such as history of unprotected sex, sexually transmitted infection or partner viral load. The association of the Cape Town site with HIV acquisition is an artefact of our sample selection, as we intentionally recruited additional HIV-seroconverters from the Cape Town and Orange Farm sites to boost seroconverter numbers. Although ethnicity of Cape Town and Johannesburg participants differed, there was no association of ethnicity with odds of HIV acquisition. The reason for the association of number of children at enrollment in the originally HIV-infected index participants with increased HIV-transmission risk to their partners is unclear, but does not relate to pregnancy during the study. Whether some female index partners may have been in the peripartum period at enrolment is unknown.

Stratification by gender was limited by sample size. In male partners who were HIV-uninfected at enrolment, presence of HLA antibodies at the early timepoint showed a trend to reduced odds of HIV acquisition, as did presence of partner-directed class 2 HLA antibodies. Surprisingly, the presence of partner-directed matching class 1 HLA antibodies in males HIV-uninfected at enrollment was significantly associated with *increased* odds of HIV acquisition on univariate analysis, albeit with a wide confidence interval, not significant in the multivariable model. In females who were HIV-uninfected at enrolment analyzed alone, no variables were significantly associated with odds of HIV acquisition, possibly due to the low number of HIV-uninfected females at enrollment in each group. Only the index partner viral load showed a trend to increased HIV acquisition risk by the originally HIV-uninfected female partner.

Following seroconversion, there were significantly more HIV-seroconverters than highly exposed persistently seronegative controls with HLA class 1 antibodies. In particular, significantly more HIV-seroconverters than highly exposed persistently seronegative controls expressed HLA-A and HLA-B antibodies. Similarly, HIV-seroconverters expressed significantly more matching class 1 HLA antibodies at the second timepoint compared to highly exposed persistently seronegative controls, particularly at the HLA-A locus. The same pattern was seen with linked transmissions, where there were significantly more linked HIV-seroconverters with class 1 HLA antibodies than highly exposed persistently seronegative controls. At the second timepoint, HIV-seroconverters also expressed more class 1 specificities *per individual* than highly exposed persistently seronegative controls. The change from timepoint 1 to timepoint 2 illustrates that HIV acquisition is a sensitizing event stimulating production of antibodies against HLA-A and HLA-B loci. This is an expected finding and is in keeping with other reports that HIV-infected individuals make anti-lymphocyte antibodies and HLA antibodies (44, 52, 61). HIV-infected patients often show polyclonal increases in gamma globulins and many viral infections result in polyclonal increases in IgG titres, often resulting in cross-reactive serological diagnostic assays (62, 63). We did not have data related to acute HIV seroconversion illness, but it may be that a feature of acute seroconversion illness includes *de novo* development of class 1 HLA antibodies in some patients which persist afterwards.

Regarding HIV acquisition as a sensitizing event for *de novo* induction of HLA antibodies, it is interesting to note, however, that the sensitization seems to be limited to class 1 rather than class 2 antibodies. Despite multiple homologies between HIV proteins and human proteins described, why HIV infection does not stimulate new class 2 antibodies in HIV-seroconverters is an intriguing question. It may be that homologies between HIV proteins and human class 2 MHC proteins result in some form of immune tolerance that limits *de novo* development of class 2 HLA antibodies. Alternatively repeated stimulation or exposure to certain HIV proteins homologous to class 2 antigens may result in blunting of the humoral immune response to class 2 antigens, such as that seen with pneumococcal polysaccharide vaccination (64).

Our data have relevance to alloimmunization strategies. Potential advantages of alloimmunization as an HIV prevention strategy includes the strong immunogenicity of HLA and avoidance of the high variability and mutability of HIV (65, 66). Potential disadvantages include the risk of induction of autoimmunity and limitations to future transplantation options in vaccinated individuals including kidney transplantation (65). Also the HLA type of the potential HIV “donor” could not be predicted, which may require incorporation of a group of multiple HLA specificities into a vector (10) if “matching” antibodies were required for protection. Our findings however suggest that there may be some benefit even from “non-matching” class 2 HLA antibodies. The benefit:risk ratio of a successful HIV vaccine would likely outweigh concerns around organ transplantation for HIV endemic areas. Multiparous women and patients who have received multiple transfusions represent natural examples of people with alloimmunity without obvious autoimmune side effects. Our data lend weight to this opinion, showing that 79% of healthy highly exposed persistently seronegative individuals express HLA antibodies with no known deleterious consequences. Additionally, 27% of healthy highly exposed persistently seronegative individuals expressed potential HLA *autoantibodies* in at least one locus, tested to two-digit resolution, without apparent consequences. It may be these antibodies are non-reactive against the individual’s HLA type tested at higher resolution, although others have also reported HLA autoantibodies in healthy individuals (67, 68), including when using high resolution HLA typing (67).

Strengths of this work include inclusion of a rare sample group, namely HIV-seroconverters followed longitudinally from prior to the date of seroconversion and afterwards. Another strength was inclusion of multiple loci for both HLA class 1 and HLA class 2 analysis, allowing a comprehensive overview. Many earlier studies have disregarded the potential influence of class 2 HLA antigens and focused solely on HLA class 1 antigens. We used highly sensitive Luminex single antigen methodology which detects even low-level antibody. We also looked specifically for complement-fixing class 2 HLA antibodies.

Weaknesses of the study include the relatively low number of HIV-seroconverters enrolled, leading to findings of borderline statistical significance that would benefit from replication in larger sample sets. Regarding complement-fixing antibodies, due to sample volume constraints, we focused on class 2 antibodies only and did not analyze complement-fixing class 1 HLA antibodies. While IgG1 and IgG3 antibodies may also fix complement, we did not investigate antibody isotypes directly and did not confirm that complement-fixing antibodies were of IgM isotype. We did not compare frequency of HLA alleles or haplotypes between groups. While HLA type and HLA antibodies may be interrelated variables, it is equally valid to interrogate either as the primary variable. We did not include antibody titre or mean fluorescence intensity in our analysis, as each individual can have many HLA antibody specificities, each with its own titre. Also, the kits vary in the number of beads per low resolution antigen, for example kits may contain HLA-A*01:01 and HLA-A*01:02 beads, each of which may give a positive result with a different titre. Antibodies of a low titre may be no less relevant than antibodies of a high titre and information may be lost by summarizing an average or maximum titre antibody per locus. Hence we chose to express antibodies in a binary fashion as present or absent at the 2-digit resolution rather than add the complexity of multiple titres per individual per locus, that is we performed a qualitative rather than quantitative analysis. We did not perform eplet analysis and cannot exclude that an antibody we have labelled as reactive with two individual HLA specificities may be cross-reacting with one smaller epitope contained within the two larger HLA proteins. While potential concerns of Luminex based methods include that of being overly sensitive or containing a mixture of intact and denatured HLA proteins, our single antigen kits have been shown to contain only intact HLA trimers, at least for class 1 (69),, with clinical relevance of such antibodies best assessed through correlation with clinical outcome, unusually feasible in our rare cohort.

As most of our partners who were HIV-uninfected at enrolment were male, our reported frequencies of HLA antibodies may be lower than that of the general population, or of multiparous females in particular. It is interesting that we found such high frequencies of HLA antibodies in a predominantly male study cohort. Future analyses of HLA antibodies or alloimmunization strategies should be specifically powered to analyze males and females separately. Multiple host factors may influence HIV susceptibility [reviewed in (70)] and we could not control for all potential confounders. Our study was an epidemiological analysis and further mechanistic studies are warranted to probe interaction between HLA antibodies and HIV infection. Such mechanistic studies have already shown proof of concept that antibodies against heterologous proteins in the viral envelope do affect viral neutralization *in vitro* (71).

## Conclusion

In summary, we have shown that more highly exposed persistently seronegative individuals had class 2 HLA antibodies than individuals who later acquired HIV infection. The presence of class 2 HLA antibodies was significantly associated with reduced odds of HIV acquisition, even after controlling for HIV-infected index partner viral load and other risk factors. These epidemiological data support the hypothesis that class 2 HLA antibodies may be partially protective against HIV acquisition; and re-invigorate the debate regarding potential merits of an alloimmunization approach against HIV, incorporating HLA class 2 alleles as vaccine antigens. A potential approach to explore the effect of class 2 HLA antibodies on HIV acquisition could include passive vaccination approaches which would be unlikely to harbor long-term risk of autoimmunity.

In terms of antibody prevalence, 79% percent of highly exposed, persistently seronegative healthy individuals harbored any HLA antibodies and 27% harbored HLA autoantibodies to at least one locus. HLA antibodies, including HLA autoantibodies, thus occur naturally without deleterious consequences. HLA antibodies are present in pharmaceutical preparations of intravenous immune globulins prepared from pooled blood donors [discussed in (67)] with no documented harm; while active immunization of pregnant women with partner white blood cells has previously been used as infertility treatment (72, 73). Fear of induction of autoantibodies remains the primary concern of an alloimmunization strategy and whether such antibodies are indeed harmful in the non-transplant setting warrants directed investigation.

## Data Availability Statement

The original contributions presented in the study are included in the article/**Supplementary Material**. Further inquiries can be directed to the corresponding author.

## Ethics Statement

The studies involving human participants were reviewed and approved by Human Research Ethics Committee of the University of the Witwatersrand. The patients/participants provided their written informed consent to participate in this study.

## Author Contributions

MS: lead author, conceptualization, sample retrieval, data retrieval, data analysis, and wrote and revised original draft. NM: sample acquisition and critical review of manuscript. SM: laboratory work and critical review of manuscript. DA: formative discussions and critical review of manuscript. RM: sample acquisition and retrieval, data retrieval, and critical review of manuscript. JL: sample acquisition and retrieval, data retrieval, and critical review of manuscript. XH: data retrieval and critical review of manuscript. HRa laboratory work and critical review of manuscript. SD-M: sample acquisition and critical review of manuscript. DC: sample acquisition and critical review of manuscript. KO: data analysis and critical review of manuscript. LM: study planning and critical review of manuscript. CT: study planning and critical review of manuscript. DS: laboratory work, sample retrieval, data retrieval, study planning, and critical review of manuscript. All authors attest they meet the ICMJE criteria for authorship. All authors contributed to the article and approved the submitted version.

## Funding

This work was partly funded through contributions to MS from the National Health Laboratory Services Research Trust, a Discovery Foundation Academic Fellowship Award and a South African Medical Association PhD Supplementary Scholarship. The Partners trial was funded by the University of Washington. CT is a South African Research Chair in HIV Vaccine Translational Research hosted by the University of the Witwatersrand, funded by the Department of Science and Innovation and the National Research Foundation of South Africa (84177). Funders had no role in data collection, analysis report writing or decision to submit for publication.

## Conflict of Interest

The authors declare that the research was conducted in the absence of any commercial or financial relationships that could be construed as a potential conflict of interest.

## Publisher’s Note

All claims expressed in this article are solely those of the authors and do not necessarily represent those of their affiliated organizations, or those of the publisher, the editors and the reviewers. Any product that may be evaluated in this article, or claim that may be made by its manufacturer, is not guaranteed or endorsed by the publisher.
