# Supplementary figures and images for: Alloimmunity to Class 2 Human Leucocyte Antigens May Reduce HIV-1 Acquisition – A Nested Case-Control Study in HIV-1 Serodiscordant Couples
Source: Front Immunol. 2022 Mar 24;13:813412. doi: 10.3389/fimmu.2022.813412 (PMC8987441; doi:10.3389/fimmu.2022.813412)

**
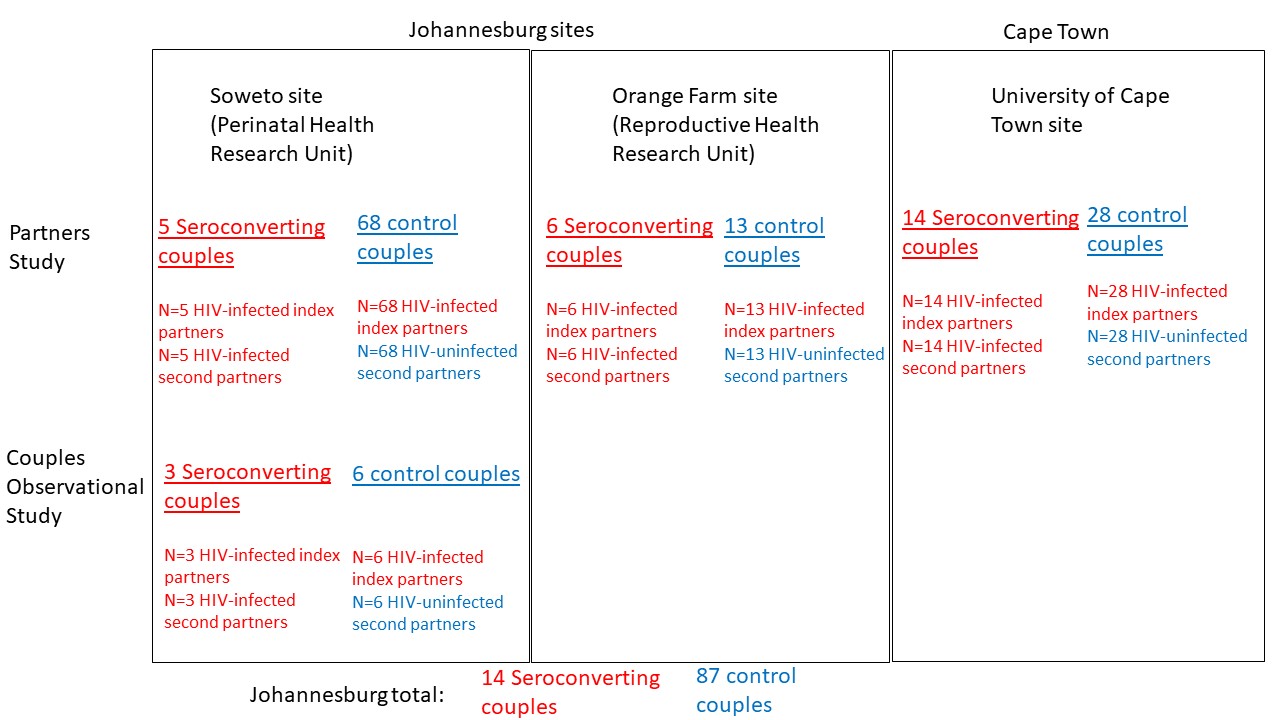
**

**Supplementary Figure 1**

**Schematic representation of participant enrollment sites**

Supplement: Supplementary file 1 [file DataSheet_1.docx]
