# Supplementary material for: Alloimmunity to Class 2 Human Leucocyte Antigens May Reduce HIV-1 Acquisition – A Nested Case-Control Study in HIV-1 Serodiscordant Couples
Source: Front Immunol. 2022 Mar 24;13:813412. doi: 10.3389/fimmu.2022.813412 (PMC8987441; doi:10.3389/fimmu.2022.813412)
Supplement: Supplementary file 2 [file DataSheet_2.docx]

**Supplementary Tables**

**Supplementary Table 1. Homologies between human proteins with HIV proteins**

| **Host protein** | | **HIV protein** | | **Reference** |
| --- | --- | --- | --- | --- |
| **Homologies with gp120** | | | | |
| HLA class 2 beta chain HLA-DR, HLA-DP, HLA-DQ) aa 142-151 | VVSTGLIQNG | gp120  (aa254-263) | VVSTQLLNG | [40][41][42] |
| HLA DP class 2 beta chain (aa 179-188) | VVSTNLIRNG | Gp120  (aa 254-263) | VVSTQLLNG | [43][25] |
| HLA-DR alpha chain (aa 28-40) | EEHVIIQAEFYLN |  | EEVVIRSANFTDN | [42] |
| HLA class 2 beta chain |  | Gp120  (aa 261-270) |  | [44] |
| HLA-DR β chain 3^rd^ hypervariable loop (aa 69-81) |  | Gp120 carboxy terminus | TKAKARRVVEREKR | [45] |
| CD4 (aa 60-64) | SLWDQ | Gp120  (aa 110-114) | SLWDQ | [43] |
| HLA class 1 alpha heavy chain (HLA-A, HLA-B, HLA-c) antigen binding site, as found on activated rather than resting T cells, not associated with β2 microglobulin | KYKY  ELYKYK  KYKR (aa 66-69) and RKLR (aa 79-82) | Gp120  C5 domain  (aa 490-492 and 505-508) | KYK and KAKR (two non-contiguous stretches flanking a hydrophobic region involved in gp41 binding) | [45][25][46][47] |
| Chemokines (MIP-1alpha, MIP-1beta, RANTES, SDF-1) 40’s loop |  | Gp120  V3 loop |  | [48] criticized in [64] |
| **Homologies with gp41** | | | | |
| Amino terminus HLA class 2 beta chain (HLA-DR, HLA-DP, HLA-DQ) (aa 19-25) | NGTERVR | Carboxy terminus of Gp41 (aa 838-844) | EGTDRVI | [49][50] |
| HLA-DR alpha chain (aa169-183) | VEHWGLDQPL | Gp41 (aa 583-599) | VERYLKDQQL | [42] |
| **Homologies with nef** | | | | |
| HLA class 2 |  | HIV nef |  | [51] |

aa amino acid position, Gp glycoprotein, in second and fourth column each amino acid is represented by a letter according to standard nomenclature

**Supplementary Table 2. Study and site of enrolment**

| **Cohort** | **Site** | **HIV-seroconverting couples** | | **Highly exposed persistently seronegative control couples** | |
| --- | --- | --- | --- | --- | --- |
|  |  | **Number of couples** | **% of seroconverter couples** | **Number of couples** | **% of control couples** |
| COS | Soweto, Johannesburg | 3 | 10.7 | 6 | 5.2 |
| Partners | Soweto, Johannesburg | 5 | 17.9 | 68 | 59.1 |
|  | Cape Town | 14 | 50.0 | 28 | 24.3 |
|  | Orange Farm, Johannesburg | 6 | 21.4 | 13 | 11.3 |
|  | **Total** | **28** | **100.0** | **115** | **100.0** |

**Supplementary Table 3. Example of antibody specificities in five highly exposed persistently seronegative controls**

|  | **CONTROLS** | | | |
| --- | --- | --- | --- | --- |
|  | **Class 1 antibodies (timepoint 1)** | **Class 1 antibodies (timepoint 2)** | **Class 2 antibodies (timepoint 1)** | **Class 2 antibodies (timepoint 2)** |
| 1 | B*73:01 | B*73:01 | DRB1*14:04 | DRB1*14:04 |
|  | B*08:01 | B*08:01 | DRB1*08:01 | DRB1*08:01 |
|  |  |  | DRB1*08:02 | DRB1*08:02 |
|  |  |  | DRB1*12:02 | weak DRB1*12:02 |
|  |  |  | DRB1*12:01 | weak DRB1*12:01 |
| 2 | A*26:01 | A*26:01 | DQA1*01:01 | DQA1*01:01 |
|  |  | A*25:01 | DQA1*01:02 | DQA1*01:02 |
|  |  | A*33:01 | DQA1*01:03 | DQA1*01:03 |
|  |  | A*66:02 | DQA1*01:04 | DQA1*01:04 |
|  |  |  | DQB1*05:01 | DQB1*05:01 |
|  |  |  | DQB1*05:02 | DQB1*05:02 |
|  |  |  | DQB1*05:03 | DQB1*05:03 |
|  |  |  | DQB1*06:01 | DQB1*06:01 |
|  |  |  | DQB1*06:02 | DQB1*06:02 |
|  |  |  | DQB1*06:03 | DQB1*06:03 |
|  |  |  | DQB1*06:04 | weak DQB1*06:04 |
| 3 | A*03:01 | A*03:01 | DRB1*08:01 | DRB1*08:01 |
|  |  | A*66:02 | DQA1*01:01 | DQA1*01:01 |
|  |  |  | DQA1*01:02 | DQA1*01:02 |
|  |  |  | DQA1*01:03 | DQA1*01:03 |
|  |  |  | DQA1*01:04 | DQA1*01:04 |
|  |  |  |  | DQA1*06:01 |
|  |  |  | DQB1*05:01 | DQB1*05:01 |
|  |  |  | DQB1*05:02 | DQB1*05:02 |
|  |  |  | DQB1*05:03 | DQB1*05:03 |
|  |  |  | DQB1*06:01 |  |
|  |  |  | DQB1*06:02 | DQB1*06:02 |
|  |  |  | DQB1*06:03 | DQB1*06:03 |
|  |  |  | DQB1*06:04 | DQB1*06:04 |
| 4 | A*11:02 | A*11:02 | - | - |
|  | A*25:01 | A*25:01 |  |  |
|  | B*37:01 weak | B*55:01 |  |  |
| 5 | - | . | DRB1*15:01 | DRB1*15:01 |
|  |  |  |  | DRB1*15:02 weak |

*In these highly exposed persistently seronegative controls, individual 1 show reproducible class 1 and class 2 specificities, with a decrease in mean fluorescent intensity of HLA-DRB1*12:01 and HLA-DRB1*12:02 antibodies from strong at the first timepoint to weak at the second timepoint. Individual 2 shows a consistent HLA-A*26:01 antibody, with development of new HLA-A*25:01, HLA-A*33:01 and HLA-A*66:02 antibodies at the second timepoint. Individual 2’s class 2 specificities were highly reproducible over time, with only a weakened intensity of HLA-DQB1*06:04 antibody from strong to weak. For individual 3, the HLA-A*03:01 antibody was reproducible, with new development of an HLA-A*66:02 antibody at the second timepoint. The class 2 antibodies were highly comparable at the two timepoints, with new development of a HLA-DQA1*06:01 specificity and loss of a HLA-DQB1*06:01 specificity. In individual 4, HLA-A*11:02 and HLA-A*25:01 recurred at both timepoints, while a weak HLA-B*37:01 antibody present at the first timepoint did not recur, and a new HLA-B*55:01 antibody was noted at the second timepoint. Individual 4 had no class 2 antibodies. Individual 5 had no class 1 antibodies. In individual 5, HLA-DRB1*15:01 antibody was present at both timepoints and weak HLA-DRB1*15:02 antibody only at the second timepoint.*

**Supplementary Table 4. Example of antibody specificities in five HIV-seroconverters**

|  | **SEROCONVERTERS** | | | |
| --- | --- | --- | --- | --- |
|  | **Class 1 antibodies (timepoint 1)** | **Class 1 antibodies (timepoint 2)** | **Class 2 antibodies (timepoint 1)** | **Class 2 antibodies (timepoint 2)** |
| 6 | - | A*23:01 weak | - | DRB3*01:01 |
|  |  | A*24:03 weak |  |  |
|  |  | A*24:02 weak |  |  |
|  |  | C*04:01 weak |  |  |
|  |  | A*02:02 weak |  |  |
|  |  |  |  |  |
| 7 | A*24:02 | A*24:02 | - | - |
|  | A*24:03 | B*15:12 |  |  |
|  |  | B*82:02 |  |  |
|  |  |  |  |  |
| 8 | - | A*24:02 | DRB1*13:05 | DRB1*13:05 |
|  |  | A*24:03 |  |  |
|  |  | B*27:05 |  |  |
|  |  | B*15:16 |  |  |
|  |  | B*54:01 |  |  |
|  |  | B*58:01 |  |  |
|  |  | B*39:01 |  |  |
| 9 | A*30:01 | A*30:01 | - | - |
|  | A*31:01 weak | A*31:01 |  |  |
|  | B*46:01 | A*43:01 |  |  |
|  |  | B*44:02 weak |  |  |
|  |  | B*45:01 |  |  |
| 10 | - | A*03:01 | - | DRB1*07:01 |
|  |  | A*11:01 |  |  |
|  |  | possible A*30:01 |  |  |
|  |  | possbile A*36:01 |  |  |
|  |  | possible A*11:02 |  |  |
|  |  | possible A*01:01 |  |  |

*In HIV-seroconverters, individual 6 had no HLA-A antibodies at the first timepoint (prior to HIV seroconversion) but developed weak HLA-A*02:02, HLA-A*23:01, HLA-A*24:02, HLA-A*24:03 and HLA-C*04:01 antibodies at the second timepoint (after HIV-seroconversion). There were no class 2 antibodies at either timepoint. Individual 7 showed a reproducible HLA-A*24:02 antibody but loss of a HLA-A*24:03 antibody and development of HLA-B*15:12 and HLA-B*82:02 antibodies. Individual 7 had no class 2 antibodies at either timepoint. Individual 8 had no HLA antibodies at the first timepoint but developed HLA-A*24:02, HLA-A*24:03, HLA-B*27:05, HLA-B*15;16, HLA-B*54:01, HLA-B*58:01 and HLA-B*39:01 antibodies at the second timepoint. There was a reproducible class 2 HLA-DRB1*13:05 antibody at both timepoints. In individual 9, There was a reproducible HLA-A*30:01 antibody and a weak HLA-A*31:01 antibody that became stronger at the second timepoint. There was disappearance of a HLA-B*46:01 specificity and development of new HLA-A*43:01, HLA-B*45:01 and weak HLA-B*44:02 specificities. There were no class 2 antibodies at either timepoint. In couple 10 we noted new development of HLA-A*02:01, HLA-A*11:01 and some other possible HLA-A specificities. “Possible” specificities were not considered further in the statistical analysis. Only “strong” and “weak” specificities were included in the chi square analyses. There was new development of a HLA-DRB1*07:01 specificity at the second timepoint*.

**Supplementary Table 5. List of complement-fixing antibodies in highly exposed persistently seronegative controls and HIV-seroconverters at the first timepoint**

“Control couples” represent highly exposed persistently seronegative individuals. “Seroconverting couples” represent individuals who acquired HIV.

**Supplementary Table 6. Risk factors for HIV acquisition by males who were HIV-uninfected at enrolment**

(n=84 highly exposed persistently seronegative male controls and 21 male HIV-seroconverters)

| Male seroconverters versus male highly exposed persistently seronegative controls | | | | |
| --- | --- | --- | --- | --- |
|  | **Univariate** |  | **Multivariate** |  |
| **Variable** | **OR (95% CI)** | **p value** | **OR (95% CI)** | **p value** |
| Cohort: Cos vs Partners | 1.334 (0.132-13.51) | 0.8075 |  |  |
| Ethnicity: Sotho vs Other | 2.222 (0.363-13.61) | 0.3881 |  |  |
| Zulu vs Other | 3.333 (0.696-15.96) | 0.1320 |  |  |
| Site: Cape Town vs Johannesburg | 3.195 (1.188-8.596) | **0.0214*** | 3.218 (1.136-9.117) | **0.0278** |
| Number of children at enrolment | 0.774 (0.518-1.159) | 0.2135 |  |  |
| Ever unprotected sex: No vs Yes | 0.977 (0.375-2.549) | 0.9625 |  |  |
| Proportion unprotected sex | 0.705 (0.106-4.684) | 0.7177 |  |  |
| Number unprotected sex | 0.929 (0.708-1.219) | 0.5930 |  |  |
| No. of no condom sex acts | 0.993 (0.967-1.020) | 0.6225 |  |  |
| Interval between 2 timepoints (mo) | 1.030 (0.942-1.126) | 0.5202 |  |  |
| Chlamydia: Positive vs Negative | 1.027 (0.108-9.718) | 0.9818 |  |  |
| Trichomonas: Positive vs Negative | 3.441 (0.704-16.81) | 0.1268 |  |  |
| Genital ulcers: Yes vs No | 1.128 (0.364-3.500) | 0.8342 |  |  |
| Any STI: Positive vs Negative | 3.850 (0.930-15.94) | **0.0629** |  |  |
| Earliest Log VL | 1.971 (0.944-4.119) | **0.0710** | 1.731 (0.801-3.744) | 0.1631 |
| Max Log VL | 1.949 (1.026-3.700) | **0.0414** |  |  |
| Median Log VL | 1.650 (0.870-3.128) | 0.1254 |  |  |
| Any class 1 HLA Abs first timepoint: yes vs no | 1.100 (0.419-2.886) | 0.8464 |  |  |
| Number of class 1 specificities first timepoint | 1.115 (0.910-1.367) | 0.2934 |  |  |
| Any class 2 HLA abs first timepoint: yes vs no | 0.346 (0.115-1.038) | **0.0584** |  |  |
| Number of DRB1 and DRB345 specificities first timepoint | 0.670 (0.280-1.602) | 0.3676 |  |  |
| Matching class 1 first timepoint: yes vs no | 6.333 (1.357-29.55) | **0.0188*** |  |  |
| Matching class 2 first timepoint: yes vs no | 0.149 (0.018-1.196) | **0.0732** |  |  |

STI sexually transmitted infection, VL viral load

**Supplementary table 7: Risk factors for HIV acquisition by females who were HIV-uninfected at enrolment**

(n=31 highly exposed persistently seronegative female controls and 7 female HIV-seroconverters)

| Female HIV-seroconverters versus female highly exposed persistently seronegative controls | | |
| --- | --- | --- |
|  | **Univariate** |  |
| **Variable** | **OR (95% CI)** | **P value** |
| Cohort: COS vs Partners | 3.733 (0.492-28.33) | 0.2026 |
| Ethnicity: Sotho vs Other | 0.444 (0.034-5.880) | 0.5383 |
| Zulu vs Other | 1.143 (0.170-7.693) | 0.8908 |
| Site: Cape Town vs Johannesburg | 3.125 (0.547-17.84) | 0.1999 |
| Number of children at enrolment | 0.696 (0.316-1.532) | 0.3679 |
| Ever unprotected sex: No vs Yes | 0.634 (0.106-3.802) | 0.6176 |
| Proportion unprotected sex | 0.436 (0.008-25.04) | 0.6880 |
| Number unprotected sex | 1.039 (0.591-1.826) | 0.8939 |
| Number of no condom sex acts | 0.967 (0.876-1.067) | 0.5020 |
| Number of class 1 specificities first timepoint | 0.982 (0.912-1.058) | 0.6337 |
| Any class 1 HLA Abs first timepoint: yes vs no | 0.533 (0.097-2.939) | 0.4704 |
| Number of DRB1 and DRB345 specificities first timepoint | 0.837 (0.424-1.652) | 0.6084 |
| Any class 2 HLA abs first timepoint: yes vs no | 0.563 (0.106-2.999) | 0.5004 |
| Matching class 1 first timepoint: yes vs no | 0.371 (0.036-3.838) | 0.4059 |
| Matching class 2 first timepoint: yes vs no | 0.778 (0.117-5.162) | 0.7947 |
| Interval between 2 timepoints (months) | 1.067 (0.920-1.237) | 0.3887 |
| Trichomonas: Positive vs Negative | 1.619 (0.220-11.89) | 0.6358 |
| Genital ulcers: Yes vs No | 0.727 (0.121-4.388) | 0.7284 |
| Any STI: Positive vs Negative | 1.333 (0.184-9.660) | 0.7758 |
| Earliest Log VL | 3.494 (0.765-15.95) | 0.1063 |
| Max Log VL | 2.188 (0.603-7.947) | 0.2340 |
| Median Log VL | 4.331 (0.891-21.07) | **0.0693** |

No multivariate analysis was performed as no factors had p values below 0.05 in univariate analysis. STI sexually transmitted infection. VL viral load.

**Supplementary Table 8: Risk factors for HIV transmission from index partner who was HIV-infected at enrolment**

(n=115 HIV-infected index partners to highly exposed persistently seronegative controls and 19 HIV-infected index partners to linked HIV-seroconverters)

| HIV-infected Index Partners – HIV-transmitters versus HIV-non-transmitters | | | | |
| --- | --- | --- | --- | --- |
|  | **Univariate** |  | **Multivariate** |  |
| **Variable** | **OR (95% CI)** | **p value** | **OR (95% CI)** | **p value** |
| Gender: Females vs Males | 1.033 (0.344-3.107) | 0.9537 |  |  |
| Enrolment age (years) | 1.011 (0.951-1.074) | 0.7333 |  |  |
| Cohort: Cos vs Hsv | 3.406 (0.774-14.99) | 0.1050 |  |  |
| Ethnicity: Sotho vs Other | 1.706 (0.220-13.24) | 0.6096 |  |  |
| Xhosa vs Other | 2.836 (0.572-14.06) | 0.2019 |  |  |
| Zulu vs Other | 3.782 (0.697-20.52) | 0.1232 |  |  |
| Site: Cape Town vs Johannesburg | 2.260 (0.827-6.176) | 0.1120 |  |  |
| Number of children at enrolment | 0.663 (0.418-1.051) | **0.0805** | 0.576 (0.329-1.007) | **0.0528** |
| Ever unprotected sex: No vs Yes | 0.148 (0.033-0.669) | **0.0131*** | 0.088 (0.018-0.440) | **0.0031*** |
| Proportion unprotected sex | 0.828 (0.078-8.814) | 0.8756 |  |  |
| Number unprotected sex | 0.972 (0.850-1.111) | 0.6776 |  |  |
| No. of no condom sex acts | 0.992 (0.970-1.013) | 0.4469 |  |  |
| Contraception injection | 1.091 (0.408-2.919) | 0.8624 |  |  |
| Genital ulcers: Yes vs No | 1.653 (0.595-4.587) | 0.3347 |  |  |
| Any STI: Positive vs Negative | 1.152 (0.369-3.595) | 0.8081 |  |  |
| Earliest Log VL | 3.813 (1.563-9.305) | **0.0033*** |  |  |
| Max Log VL | 2.098 (1.062-4.143) | **0.0328*** |  |  |
| Median Log VL | 2.482 (1.182-5.212) | **0.0163*** | 3.038 (1.282-7.200) | **0.0116*** |

STI sexually transmitted infection, VL viral load

**Supplementary Table 9: Risk factors for HIV transmission from male index partners who were HIV-infected at enrolment**

(n=31 male index partners of highly exposed persistently seronegative individuals and 5 male index partners of linked HIV-seroconverters)

| Male HIV-infected Index Partners – HIV transmitters versus HIV-non-transmitters | | |
| --- | --- | --- |
|  | **Univariate** |  |
| **Variable** | **OR (95% CI)** | **p value** |
| Enrolment age (years) | 0.947 (0.817-1.098) | 0.4688 |
| Cohort: Cos vs Partners | 6.222 (0.725-53.37) | **0.0955** |
| Site: Cape Town vs Johannesburg | 2.778 (0.376-20.50) | 0.3164 |
| Number of children at enrolment | 0.695 (0.278-1.738) | 0.4367 |
| Ever unprotected sex: No vs Yes | 0.267 (0.027-2.665) | 0.2604 |
| Proportion unprotected sex | 0.526 (0.004-72.73) | 0.7986 |
| Number unprotected sex | 0.952 (0.693-1.307) | 0.7597 |
| No. of no condom sex acts | 0.980 (0.906-1.059) | 0.6053 |
| Genital ulcers: Yes vs No | 2.286 (0.316-16.51) | 0.4126 |
| Any STI: Positive vs Negative | 1.278 (0.112-14.59) | 0.8435 |
| Earliest Log VL | 7.945 (0.927-68.09) | **0.0586** |
| Max Log VL | 2.925 (0.638-13.41) | 0.1672 |
| Median Log VL | 6.810 (0.945-49.07) | **0.0569** |

STI sexually transmitted infection, VL viral load

**Supplementary Table 10: Univariate risk factors for HIV transmission from female index partners who were HIV-infected at enrolment**

(n=84 female HIV-infected index partners of highly exposed persistently seronegative individuals and 14 female HIV-infected index partners of HIV-seroconverters)

| Female HIV-infected index partners - HIV-transmitters versus HIV-non-transmitters | | | | |
| --- | --- | --- | --- | --- |
|  | **Univariate** |  | **Multivariate** |  |
| **Variable** | **OR (95% CI)** | **p value** | **OR (95% CI)** | **p value** |
| Enrolment age (years) | 1.026 (0.960-1.096) | 0.4505 |  |  |
| Cohort: Cos vs Partners | 2.077 (0.201-21.51) | 0.5400 |  |  |
| Ethnicity: Sotho vs Other | 0.607 (0.050-7.415) | 0.6960 |  |  |
| Xhosa vs Other | 1.653 (0.310-8.815) | 0.5563 |  |  |
| Zulu vs Other | 2.000 (0.322-12.41) | 0.4568 |  |  |
| Site: Cape Town vs Johannesburg | 2.114 (0.659-6.775) | 0.2080 |  |  |
| Number of children at enrolment | 0.644 (0.374-1.108) | 0.1121 | 0.558 (0.302-1.030) | **0.0621** |
| Ever unprotected sex: No vs Yes | 0.103 (0.013-0.820) | **0.0318*** | 0.088 (0.017-0.451) | **0.0036*** |
| Proportion unprotected sex | 0.960 (0.063-14.54) | 0.9767 |  |  |
| Number unprotected sex | 0.977 (0.841-1.134) | 0.7556 |  |  |
| No. of no condom sex acts | 0.993 (0.972-1.015) | 0.5416 |  |  |
| Genital ulcers: Yes vs No | 1.474 (0.447-4.862) | 0.5243 |  |  |
| Any STI: Positive vs Negative | 1.104 (0.303-4.025) | 0.8806 |  |  |
| Earliest Log VL | 3.164 (1.165-8.596) | **0.0239*** | 4.683 (1.677-13.08) | **0.0032*** |
| Max Log VL | 1.930 (0.909-4.096) | **0.0870** |  |  |
| Median Log VL | 2.007 (0.903-4.458) | **0.0873** |  |  |

STI sexually transmitted infection, VL viral load
